# Effects of higher versus lower protein intake in critically ill patients: a systematic review and meta-analysis

**DOI:** 10.3389/fnut.2026.1900749

**Published:** 2026-09-25

**Authors:** Shige Li, Yusheng Hou, Xinyu Li, Hongyu Jin, Leilei Fu, Shengchen Wang, Xiangrui Li, Meng Sun, Haijia Hou, Dandan Song, Bing Dai, Hongwen Zhao, Wei Wang, Jian Kang, Wei Tan

**Affiliations:** 1 Department of Respiratory and Critical Care Medicine, The First Hospital of China Medical University, Shenyang, China; 2 School of Medicine, Zhejiang University, Hangzhou, China; 3 Department of Endocrinology, The First Hospital of China Medical University, Shenyang, China; 4 Department of Pathophysiology, College of Basic Medical Sciences, Jinzhou Medical University, Jinzhou, China

**Keywords:** acute kidney injury, critical illness, meta analysis, protein, rehabilitation

## Abstract

**Background:**

The optimal protein dose for critically ill patients remains uncertain, and emerging evidence suggests that the effects of higher protein intake may differ across clinically relevant subgroups.

**Methods:**

PubMed, Embase, Web of Science, Scopus, and the Cochrane Library were searched for randomized controlled trials (RCTs) evaluating protein delivery in critical illness adults (up to July 2026). Higher and lower protein groups were defined according to each trial’s original allocation. Primary outcome was all-cause mortality. Secondary outcomes included mechanical ventilation (MV) duration, Intensive care unit (ICU) length of stay (LOS), muscle outcomes, health-related quality of life (HRQoL), biochemical outcomes, and adverse events. Results were reported as risk ratios (RRs) or mean difference (MD) and 95% confidence intervals (CIs). Trial Sequential Analysis (TSA) was performed to control for random error, and the certainty of evidence was rated using the GRADE framework.

**Results:**

Thirty three RCTs involving 11,622 participants were included. No significant differences were observed between higher and lower protein groups for all-cause mortality (RR 1.02, 95% CI 0.97 to 1.08), MV duration (MD −0.48, 95% CI −1.08 to 0.13), or ICU LOS (MD −0.18, 95% CI −0.86 to 0.51), with TSA confirming these null findings. Some muscle metrics favored the higher protein group; however, there was a trend towards poorer HRQoL (SMD −0.12, 95% CI −0.24 to 0.00). The higher protein group showed improved nitrogen balance (MD 5.58, 95% CI 2.89 to 8.27) and higher serum albumin levels (SMD 0.18, 95% CI 0.07 to 0.29), but increased urea (MD 2.06, 95% CI 1.49 to 2.64) and blood urea nitrogen concentrations (MD 2.94, 95% CI 1.32 to 4.56). Sensitivity analyses restricted to 27 isocaloric-design trials yielded consistent results. Subgroup analyses revealed that higher protein intake was linked to increased mortality in patients with acute kidney injury (RR 1.46, 95% CI 1.14 to 1.87); however, higher protein intake combined with rehabilitation interventions was associated with reduced mortality (RR 0.68, 95% CI 0.50 to 0.93), shorter MV duration (MD -1.45, 95% CI −2.61 to −0.29), and beneficial muscle effects.

**Conclusion:**

Higher protein intake was not associated with better clinical outcomes. While it may increase mortality in patients with AKI, combining it with physical rehabilitation yields survival and clinical benefits, warranting confirmation in future factorial RCTs.

**Systematic review registration:**

https://www.crd.york.ac.uk/PROSPERO/view/CRD420251109618, identifier [CRD420251109618].

## 1 Introduction

Critical illness significantly disrupts the body’s homeostatic balance, initiating complex metabolic changes that adversely affect patient recovery and quality of life (QoL) (1). Nutritional support is a fundamental aspect of critical care in the intensive care unit (ICU), aimed at preserving or restoring nutritional status and muscle function, and promoting healing and faster recovery from illness (2, 3). Within this context, protein is especially vital, providing essential substrate for tissue repair, immune competence, and maintenance of organ function (2, 3).

The optimal dosing of protein supplementation remains one of the most contentious issues in critical care nutrition. Recent large randomized controlled trials (RCTs) have shown no general benefit of higher-dose protein; however, uncertainties persist regarding subgroup effects, dose thresholds, and rehabilitation interaction. International guidelines provide a broad range of recommendations (4–7) (e.g., 1.3 g/kg/day, 1.2–2.0 g/kg/day, 1 or 1.2 g/kg/day, 0.2–0.9 g/kg/day in the first week to 1.0–1.3 g/kg/day in the second week). Prior to the recent wave of large RCTs, several prospective cohort studies (8, 9) reported survival benefits and dose–response relationships with higher protein delivery, which informed these guideline recommendations. However, these recommendations are based on lower-quality evidence and lead to significant variability in clinical practice across institutions. A previous meta-analysis of 23 RCTs (*n* = 3,303) (10) concluded that higher protein intake does not significantly affect general clinical outcomes in most critically ill patients but may increase mortality in those with acute kidney injury (AKI). Subsequent high-quality RCTs have further refined this understanding: the NUTRIREA-3 study (11) (*n* = 3,044) found that early calorie and protein restriction did not decrease mortality but was associated with faster recovery and fewer complications; the PRECISe study (12) (*n* = 935) reported that high enteral protein intake (2.0 g/kg/day) fails to improve functional outcomes and worsens health-related quality of life(HRQoL); and the TARGET protein trial (13) (*n* = 3,397) showed no benefit of increased enteral protein on 90-day hospital-free survival.

By integrating data from RCTs, this meta-analysis rigorously evaluates the impact of higher protein intake on clinical outcomes, muscle outcomes, biochemical outcomes, HRQoL, and adverse events in critically ill patients. Furthermore, we explore whether the effects of high protein doses differ across specific ICU subgroups (e.g., patients with AKI), and whether these outcomes are modulated by concurrent physical rehabilitation.

## 2 Methods

This study adhered to the Preferred Reporting Items for Systematic Reviews and Meta-Analyses (PRISMA) 2020 statement (14) (Supplementary Table S1) and the methodological standards outlined in the Cochrane Handbook for Systematic Reviews of Interventions (15, 16). The study protocol was prospectively registered with PROSPERO (CRD420251109618).

### 2.1 Search strategy

Two reviewers (Li S and Hou Y) independently searched the PubMed, Embase, Web of Science, Scopus, and Cochrane Central Register of Controlled Trials databases up to July 2026. Core search terms included (“nutrition therapy” OR “protein” OR “energy” OR “feeding” etc.) AND (“critical care” OR “ICU” etc.). Full strategies are detailed in Supplementary Table S2. Reference lists of relevant studies and reviews were also screened for additional citations.

### 2.2 Inclusion and exclusion criteria

Studies were eligible if they (1) enrolled adult participants (aged ≥18 years) with critical illness; (2) were randomized controlled trials with a parallel group design comparing higher versus lower provision of protein or amino acids, as defined within each trial; (3) delivered protein via any route; and (4) reported mortality, patient-centered outcomes, or biochemical parameters. Higher and lower protein groups were defined according to each trial’s original allocation. No universal gram-per-kilogram cut-off was imposed for the primary analysis; pre-specified sensitivity analyses using thresholds of 1.2, 1.3, and 1.5 g/kg/day were additionally performed. Exclusion criteria included non-adult populations, laboratory or animal studies, non-English-language publications, and non-original study types such as commentaries, reviews, editorials, conference abstracts, case reports, brief surveys, study protocols, and duplicate publications. Studies were also excluded if they did not explicitly report target outcomes or focused on immunonutrients (e.g., glutamine, arginine, or omega-3 fatty acids). Any discrepancies between reviewers were resolved through discussion and consensus.

### 2.3 Outcomes

The primary outcome was all-cause mortality (assessed at the longest follow-up). Mortality outcomes included ICU mortality, hospital mortality, and mortality at 28, 30, 60, 90, and 180 days. Secondary outcomes included ICU length of stay (LOS), duration of mechanical ventilation (MV), muscle outcomes (the atrophy rate of thigh muscle thickness, the atrophy rate of thigh muscle cross-sectional area (CSA), the absolute change in mid-upper arm circumference [MUAC], diaphragm atrophy, and handgrip strength), HRQoL assessed using the EQ-5D-5L or SF-36, biochemical parameters (nitrogen balance, serum albumin, serum urea, serum creatinine, blood urea nitrogen [BUN]), and adverse events (feeding intolerance, diarrhea, regurgitation or vomiting, constipation, receipt of promotility agents, received renal replacement therapy [RRT], and infection in ICU). Subgroup analyses were conducted to assess the effects of higher versus lower protein delivery across distinct populations, including the general critically ill population and those with AKI. Additionally, subgroup analysis was also performed to evaluate the impact of interventions with versus without physical rehabilitation on the outcomes of interest. Outcomes with at least three studies were pooled and reported.

### 2.4 Data extraction

The two reviewers (Li S and Hou Y) independently extracted data from the included studies following the Cochrane guidelines (15). The extracted details included first author, publication year, country, study design, participant illness characteristics, sample size, intervention protocols, including prescribed protein targets and actual protein delivery as separately reported by each study, patient demographic variables (including age, sex, body mass index [BMI], Acute Physiology and Chronic Health Evaluation II [APACHE II] score, Sequential Organ Failure Assessment [SOFA] score, Glasgow Coma Scale [GCS] score, admission diagnosis, etc.), and study outcomes. For trials incorporating physical rehabilitation, we extracted: intervention type; rehabilitation grouping; devices used; timing of initiation; session frequency and duration; intensity or progression criteria; and overall intervention duration. All extracted data were cross-validated by the two teams prior to analysis. Categorical outcomes are noted as the number of patients with each outcome compared to the total in each group. For continuous outcomes, we extracted sample size along with either mean (SD) or median (IQR) values for each reported variable, converting medians into estimated means (SD) when necessary (17, 18). For results presented as images, data were extracted using GetData Graph Digitizer. Discrepancies were resolved through iterative discussions among all authors.

### 2.5 Study quality, trial sequential analysis, and certainty of evidence

The risk of bias of the included RCTs was evaluated using the Cochrane Risk of Bias Tool for Randomized RCTs (RoB-2) (16, 19), which includes five key domains: randomization, deviations from intended interventions, missing outcome data, measurement of outcomes, and selection of reported results. Each domain was classified as “low risk,” “some concerns,” or “high risk” of bias.

Trial sequential analysis (TSA) (20, 21) version 0.9.5.10 (beta software) was used to evaluate the effect of nutrition delivery, reduce random error, assess conclusion reliability, and estimate the sample size required for the meta-analysis, with the pre-specified parameters (e.g., type I and II error rates, clinically relevant effect sizes) detailed in Supplementary Table S2.

The certainty of evidence was assessed using the Grading of Recommendations Assessment, Development, and Evaluation (GRADE) framework via the GRADEpro Guideline Development Tool (GRADEpro GDT) (22, 23). Evidence was rated across five domains: risk of bias, inconsistency, indirectness, imprecision, and publication bias, with final ratings categorized as high, moderate, low, or very low.

### 2.6 Data synthesis and analysis

All meta-analyses and subgroup analyses were conducted using RevMan version 5.4.1. prescribed protein targets and actual protein delivery were analyzed separately. For descriptive purposes, sample-size-weighted mean prescribed protein targets were calculated only for studies reporting targets in g/kg/day; data reported in g/day, g/L, or as a percentage of energy were not converted or included in this calculation. Dichotomous outcomes are summarized using risk ratios (RRs). Continuous outcomes are reported as mean differences (MDs) or standardized mean differences (SMDs). Heterogeneity was quantified using the I^2^ statistic (range: 0–100%), with thresholds of 25, 50, and 75% indicating low, moderate, and high heterogeneity, respectively. A fixed-effect model was used when *I^2^* < 50%; otherwise, a random-effects model was applied. A sensitivity analysis based on energy comparability was conducted to assess the robustness of the pooled estimates. Isocaloric trials were defined as those in which both arms were prescribed the same method and magnitude of energy targeting. Publication bias was assessed by examining funnel plot asymmetry and using Egger’s regression test and Begg’s rank correlation test. Statistical significance was set at a two-tailed *p* < 0.05.

## 3 Results

### 3.1 Search results

Our systematic database search identified 22,627 potentially relevant studies. After removing duplicates, 15,043 records proceeded to title and abstract screening, of which 14,903 were excluded. The remaining 140 studies advanced to full-text review, 105 of which were excluded based on the predefined selection criteria, resulting in 33 eligible RCTs for inclusion and 2 secondary reports for AKI subgroup analysis. The search and screening process is detailed in Figure 1.

**Figure 1:**
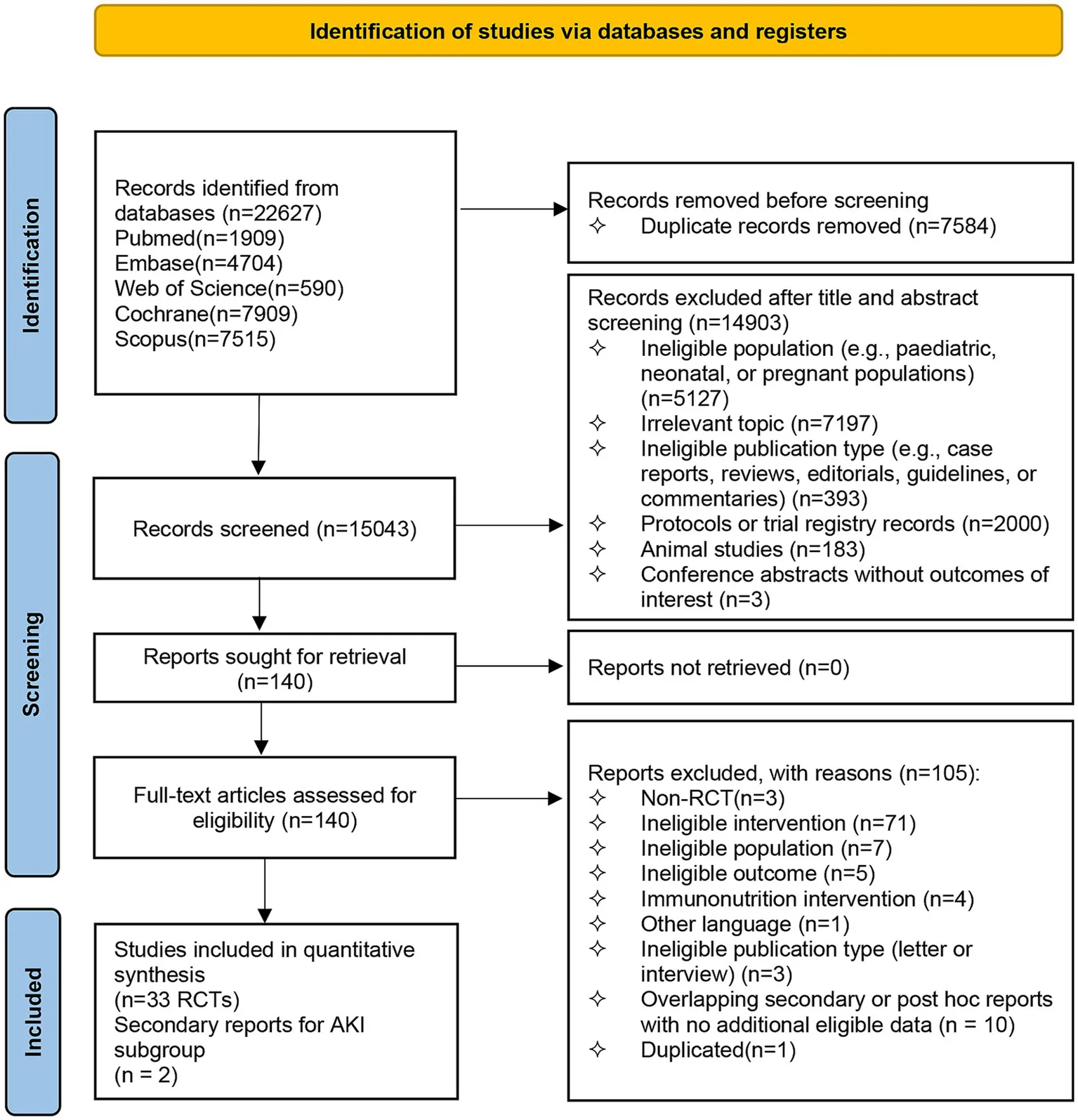
Flow diagram.

### 3.2 Characteristics of the included studies

The detailed characteristics of the included studies are given in Table 1 and Supplementary Tables S3–S6. In total, 33 RCTs (11–13, 24–53) involving 11,622 participants were included (higher-protein group: 5,777; lower-protein group: 5,845), with 2 secondary reports (54, 55) added for subgroup analyses. Sample sizes ranged from 14 to 3,397. The studies included 8 multi-center and 25 single-center RCTs conducted in medical, surgical, or mixed ICUs.

**Table 1:** Demographic details of the included RCTs.

Author, year	Country	RCT	Sample size,exp./ con(n)	Population	Intervention	Rehabilitation	EN/PN	Start intervention	Duration	Prescribed protein/energy targets		Actual protein/energy delivery		Outcomes
										Higher	Lower	Higher	Lower	
Ahmadpour et al. (2020) (24)	Iran	S	15/15	Neurocritical patients	High protein vs. conventional protein	No	EN	24–48 h after admission	5d	P: 1.5-2 g/kg/d E: Standard enteral formula	P: 0.5–1.5 g/kg/d E: Standard enteral formula	P: 1.64 ± 0.14 g/kg/d E:22.67 ± 5.23 kcal/kg/d	P: 0.54 ± 0.22 g/kg/d E:14.21 ± 5.65 kcal/kg/d	Primary: dietary factors, clinical outcome, and 28-day mortality
Azevedo et al. (2019) (25)	Brazil	S	57/63	Critically ill patients	Optimized calorie and high protein nutrition vs. Control	No	EN ± PN	Initiated after admission	14d	P: 2–2.2 g/kg/d E: Determined by indirect calorimetry	P: 1.4–1.5 g/kg/d E: 25 kcal/kg/d	P: 1.69(1.33,1.8) g/kg/d E:1139(890,1,278) kcal/d	P:1.13(0.97,1.34) g/kg/d E:1140(889,1,331) kcal/d	Primary: PCS Secondary: handgrip strength, MVD, ICU stay, ICU and hospital mortality
Azevedo et al. (2021) (26)	Brazil	S	87/94	In ICU with MV	*High-protein and early exercise vs. Control	Yes	EN ± PN	Third day of randomization	<14d	P: 2.0–2.2 g/kg/d E: Determined by indirect calorimetry	P: 1.4–1.5 g/kg/d E: Determined by indirect calorimetry	P: 1.9(1.7,2.1) g/kg/d E: 20(16,22)kcal/kg/d	P: 1.34(1.1,1.4) g/kg/d E: 19(14,21)kcal/kg/d	Primary: PCS Secondary: handgrip strength, MVD, mortality (ICU, hospital, 180-day)
Badjatia (2021) (27)	USA	S	12/13	Aneurysmal subarachnoid hemorrhage	*NMES+HPRO vs. Standard of care	Yes	EN	Within 24 h after surgery	14d	P: 1.8 g/kg/d E: by calculated	P: 1.2–1.4 g/kg/d E: by calculated	P:1.51 ± 0.47 g/kg/d E:20.04 ± 7.1 cal/kg/d	P:0.88 ± 0.36 g/kg/d E:19.8 ± 9.9 cal/kg/d	Primary: lean body mass and muscle strength Secondary: 90-day mortality
Badpeyma et al. (2025) (28)	Iran	S	28/28	Adult general ICU patients	High-protein vs. conventional-protein nutrition	NO	EN ± PN + IV AA	24–48 h after ICU admission	≤12 d	P: 2.2 g/kg/d E: 25 kcal/kg/d	P: 1.0 g/kg/d E: 25 kcal/kg/d	P: 1.67 ± 0.33 g/kg/d E: 1385 ± 245 kcal/d	P: 0.93 ± 0.10 g/kg/d E: 1248 ± 250 kcal/d	Primary: 60-d mortality; Secondary: mortality (ICU, hospital, 28-day, 60-day), MV duration, ICU/hospital stay, MAC, adverse events, biochemical indicators
Bels et al. (2024) (12)	Dutch & Belgian	M	470/465	Critically ill patients	High protein vs. standard protein	No	EN ± PN	NA	ICU stay	P: 2.0 g/kg/d E: 1.3 kcal/mL	P: 1.3 g/kg/d E: 1.3 kcal/mL	P: 1.48 ± 0.70 g/kg/d E: 19 ± 9 kcal/kg/day	P: 0.95 ± 0.44 g/kg/d E: 19 ± 9 kcal/kg/day	Primary: HRQoL and functional outcomes Secondary: mortality (30-day, 60-day, 90-day, hospital), time-to-discharge alive, adverse events, MVD, ICU/hospital stay
Bibi et al. (2025) (29)	Pakistan	S	43/45	Adult critically ill patients	2 g/kg/d vs. 1 g/kg/d	No	EN or PN	NA	NA	P: 2 g/kg/d E: ASPEN guidelines	P: 1 g/kg/d E: ASPEN guidelines	NA	NA	Primary: nitrogen balance
Bukhari et al. (2020) (30)	Indonesia	S	19/14	Adult critically ill patients	High-protein polymeric formula vs. Oligomeric formula	No	EN	24–48 h after admission	3d	P: 22.4% protein from total calorie E: 25–30 kcal/kg/d	P: 16.2% protein from total calorie E: 25–30 kcal/kg/d	P: 102.9 ± 37.5/134.0 ± 42.6 g(Non-TBI/TBI) E: same	P: 80.3 ± 29.4/93.8 ± 26.7 g(Non-TBI/TBI) E: same	Primary: WBC count, APACHE II, NUTRIC Secondary: hospital mortality
Chapple et al. (2021) (31)	Australia & New Zealand	M	58/58	In ICU with MV	High-protein vs. usual-care	No	EN	16-17 h after randomization	8d	Formula P: 100 g/L E: Isocaloric; same goal rate (1 mL/kg IBW/h)	Formula P: 63 g/L E: same	P: 1.52 ± 0.52 g/kg/d E: 19.2 ± 6.5 kcal/kgd	P: 0.99 ± 0.27 g/kg/d E: 19.6 ± 5.4 kcal/kg/d	Primary: mean daily protein delivery Secondary: duration of feeding, blinding of the intervention, tolerability of EN, biochemical indicators, mortality (ICU, 28-day, 90-day), ICU/hospital stay, days alive and ICU-free/ MV-free at day 28, HRQoL
Danielis et al. (2019) (32)	Italy	S	19/21	Critically ill patients	Protein-fortified diet vs. Standard diet	No	EN or PN	Once after admission	7d	P: 21% protein from total calorie E: REE-based	P: 16% protein from total calorie E: 20-25 kcal/kg/d	P: 118 ± 17 g/d E: 1539 ± 339 kcal/d	P: 66 ± 20 g/d E: 1692 ± 359 kcal/d	Primary: nitrogen balance Secondary: ICU-acquired skin alterations and creatinine levels, mortality after ICU discharge
Doig et al. (2015) (33)	Australia & New Zealand	M	239/235	Critically ill patients	Amino acid supplement vs. Standard care	No	EN/PN + IV AA	Within 48 h of admission	>7d	P: 2.0 g/kg/d + IV AA E: Determined by clinician	P: Usual protein, no IV AA E: Determined by clinician	P: 1.71 ± 0.08 g/kg/d E: 1145 ± 101 kcal/d	P: 0.76 ± 0.08 g/kg/d E: 1113 ± 99 kcal/d	Primary: duration of renal dysfunction Secondary: measures of renal function, mortality (ICU, hospital, 90-day)
Dresen et al. (2021) (34)	German	S	21/21	ICU patients	Intervention vs. Standard	No	EN ± PN	After≥10 d in the ICU	<28d	P: 1.8 g/kg/d E: 100% individual REE; 29 ± 7 kcal/kg/d	P: 1.2 g/kg/d E: 100% individual REE; 29 ± 9 kcal/kg/d	P: 1.5 ± 0.5 g/kg/d E: 27 ± 8.9 kcal/kg/d	P: 1.0 ± 0.5 g/kg/d E: 24.6 ± 9.8 kcal/kg/d	Primary: regain muscle mass Secondary: clinical symptoms such as pneumonia and infected wounds, ICU mortality
Elkalawy et al. (2025) (35)	Egypt & USA	M	53/49	Severe TBI patients	High-protein vs. standard-protein	NO	EN	Within 24 h	20d	P: 2.2 g/kg/d E: IC-guided	P: 1.5 g/kg/d E: IC-guided	P: 1.8 ± 0.2 g/kg/day E: time-specific, 3,605–4,199 kcal/day	P: 1.2 ± 0.4 g/kg/day E: time-specific, 3,172–3,956 kcal/day	Primary: rectus femoris muscle-CSA; Secondary: pennation angle, echogenicity, biochemical indicators, MVD, ICU stay
Ferrie et al. (2016) (36)	Australian	S	59/60	Critically ill patients receiving PN	1.2 g/kg amino acids vs. 0.8 g/kg amino acids	No	PN	17 h after randomization	10d	P: 1.2 g/kg amino acids E: 25 kcal/kg/d	P: 0.8 g/kg amino acids E: 25 kcal/kg/d	P: 7d:1.09 ± 0.22 g/kg/d E: 7d:23.1 ± 3.9 kcal/kg/d	P: 7d:0.9 ± 0.21 g/kg/d E: 7d:24.9 ± 4.2 kcal/kg/d	Primary: handgrip strength Secondary: fatigue score, muscle thickness, nitrogen balance, mortality (ICU, hospital, 180-day), ICU/hospital stay
Fetterplace et al. (2018) (37)	Australian	S	30/30	Critically ill patients	High-protein vs. Standard care	No	EN	Within 24 h	15d	P: 1.5 g/kg/d E: 25 kcal/kg/d	P: 1.0 g/kg/d E: 25 kcal/kg/d	P: 1.20 ± 0.30 g/kg/d E: 21 ± 5.2 kcal/kg/d	P: 0.75 ± 0.11 g/kg/d E: 18 ± 2.7 kcal/kg/d	Primary: protein and energy delivery (Secondary: QMLT, malnutrition, mortality 28-day, 60-day), hospital stay
Heyland et al. (2023) (38)	16 countries	M	645/656	MV patients	High-dose protein vs. Usual dose protein	No	EN or PN	Within 96 h of admission	<28d	P: ≥2.2 g/kg /d E: use published guidelines	P: ≤1.2 g/kg /d E:same	P: 1.6 ± 0.5 g/kg/d E: 14.7 ± 6.9 kcal/kg/d	P: 0.9 ± 0.3 g/kg/d E: 13.2 ± 6.4 kcal/kg/d	Primary: time-to-discharge-alive from hospital Secondary: 60-day mortality
Kagan et al. (2022) (39)	Israel	S	19/21	MV patients	*CE with protein- enriched vs. standard EN	Yes	EN	Immediately	<28d	P: Protein enriched E: measured by REE	P: EN E: measured by REE	P: 83.7 ± 31.9 g/d E: 1373 ± 531 kcal/d	P: 67.2 ± 20.2 g/d E: 1648 ± 376 kcal/d	Primary: MV days, mortality (ICU and hospital), ICU /hospital stay
Liu et al. (2024) (40)	China	S	60/60	Severe pneumo -nia patients	*High protein vs. Standard protein	Yes	EN	NA	>10d	P: 1.8 g/kg/d E: 25 kcal/kg/d	P: 1.2 g/kg/d E: 25 kcal/kg/d	NA	NA	Primary: muscle mass Secondary: nutritional status, adverse events
Miri et al. (2025) (41)	Iran	S	50/50	Critically ill patients	High-protein enteral nutrition vs. standard nutrition care	NO	EN	After randomization;	≤21 d	P: 1.5 g/kg/d E: NA	P: 1.0 g/kg/d E: NA	P: NA E: NA	P: NA E: NA	Primary: biceps brachii muscle thickness change; MUAC change. Secondary: nitrogen balance, mortality (during the study period)
Nakamura et al. (2021) (42)	Japan	S	60/57	ICU patients	*High- vs. Medium- protein, both with rehabilitation and EMS	Yes	EN ± PN	Within 48 h of admission	10d	P: 1.8 g/kg/d E: 20 kcal/kg/d	P: 0.9 g/kg/d E: 20 kcal/kg/d	P: 1.36 ± 0.80 g/kg/d E: 17.9 ± 10.1 kcal/kg/d	P: 0.72 ± 0.46 g/kg/d E: 16.2 ± 9.5 kcal/kg/d	Primary: femoral muscle volume change Secondary: PIICS, HRQoL, 28-day survival rate, ICU/hospital stay, MVD, adverse events
O’Keefe et al. (2025) (43)	USA	S	244/256	Trauma/surgical ICU patients	Early supplemental enteral protein vs. standard EN	NO	EN	24–72 h after admission	ICU stay	P: 2 g/kg/d E: EN 25 kcal/kg/d, total unclear	P: standard care E: EN 25 kcal/kg/d, total unclear	P: 1.20 ± 0.65 g/kg/d E: Higher	P: 0.60 ± 0.39 g/kg/d E: Lower	Primary: transthyretin day14–21 Secondary: ventilator-free days; hospital mortality, ICU−/hospital-free days, pneumonia, BUN, adverse events
Opoda et al. (2024) (44)	Egypt	S	36/34	MV patients	*High protein and physical exercise vs. Usual protein and care	Yes	EN ± PN + IV AA	Within 48 h of admission	NA	P: 2.0 g/kg/day E: 20–25 kcal/kg/d	P: 1.2 g/kg/day E: 20–25 kcal/kg/d	NA	NA	Primary: rectus femoris muscle CSA and thickness; Secondary: ICU stay, MVD, incidence of infection, 30-day mortality
Parasuraman et al. (2025) (45)	India	S	15/15	MV patients	High protein feed vs. Standard feed	No	EN	Within 24 h of admission	NA	P: 1.5 g/kg/day E: 25 kcal/kg/d	P: 1 g/kg/day E: 25 kcal/kg/d	NA	NA	Primary: muscle thickness &CSA Secondary: muscle echogenicity, pennation angle
Reignier et al. (2023) (11)	France	M	1,515/1,521	Adults with MV and shock	Early nutrition with standard vs. Low calorie and protein targets	No	EN or PN	Within 24 h After intubation	7d	P: 1–7d 1.0–1.3 g/kg/d E: 1–7d 25 kcal/kg/d, 8d 30 kcal/kg/d	P:1–7d 0.2–0.4 g/kg/d E:1–7d 6 kcal/kg/d, 8d 30 kcal/kg/d	P: 0.9(0.7, 1.0) g/kg/d E: 22(17.5, 24.9) kcal/kg/d	P: 0.2 (0.2, 0.3) g/kg/d E: 7.4(5.8, 9.5)kcal/kg/d	Primary: time to readiness for ICU discharge and day 90 all-cause mortality Secondary: adverse events, MV weaning
Rugeles et al. (2013) (46)	Colombia	S	40/40	Critically ill patients	Hyperproteic hypocaloric VS Isocaloric	No	EN	Within 48 h of ICU admission	7d	P: 1.7 g/kg/d E: 15 kcal/kg/d	P: NA E: 25 kcal/kg/d	P: 1.4 ± 0.1 g/kg/d E: 12 ± 0.5 kcal/kg/d	P: 0.76 ± 0.2 g/kg/d E: 14 ± 0.5 kcal/kg/d	Primary: ΔSOFA; Secondary: insulin requirements, hyperglycemia episodes, ICU stay, MVD, mortality, adverse event
Singer et al. (2007) (47)	Israel	S	8/6	ARF patients with diuresis	High vs. normal amino acid load	No	PN	NA	3d	P: 150 g/d E: 2,000 kcal/d	P: 75 g/d E: 2,000 kcal/d	NA	NA	Primary: glomerular filtration rate and nitrogen balance Secondary: hospital mortality
Summers et al. (2025) (13)	Australia & New Zealand	M	1681/1716	Critically ill patients	Augmented protein vs. Usual protein	No	EN	Within 48 h of admission	7d	Formula P: 100 g/L; E: Clinician-determined goal rate; isocaloric formula	Formula P: 63 g/L; E: Clinician-determined goal rate; isocaloric formula	P: 0.87 (0.51,1.18) g/kg/d; E: 10.9 (6.4,14.9) kcal/kg/d	P: 0.53 (0.32,0.75) g/kg/d; E: 10.5 (6.3,14.9) kcal/kg/d	Primary: number of days free of the index hospital and alive at day 90, alive at day 90, MVD, ICU /hospital stay, incidences of tracheostomy insertion and new kidney replacement therapy, hospital discharge destination
Uyar et al. (2023) (48)	Turkey	S	25/24	MV patients	High protein vs. Standard protein	No	EN	Reach the target within four days	ICU stay	P: 2.0 g/kg/d E: 25 kcal/kg/d	P: 1.2 g/kg/d E: 25 kcal/kg/d	NA	NA	Primary: diaphragm muscle thickness, correlation of diaphragm thickness fraction with rectus femoris muscle thickness, Secondary: 28-day mortality
van Zanten et al. (2018) (49)	Netherlands & France & Belgium	M	22/22	Overweight patients	Very high intact-protein vs. standard high protein	No	EN ± PN	Within 48 h of admission	>5d	Formula P: 8 g/ 100 mL E: 25 kcal/kg IBW/d	Formula P: 5 g/ 100 ml E: 25 kcal/kg IBW/d	P: 1.2 ± 0.74 g/kg/d E: 15 ± 9.3 kcal/kg/d	P: 0.7 ± 0.43 g/kg/d E: 13.8 ± 8.6 kcal/kg/d	Primary: protein intake Secondary: energy intake, amino acid levels, mortality (ICU and 28-day), adverse events
Veldsman et al. (2025) (50)	South Africa	S	25/25	Surgical ICU patients;	*Standard nutrition + Intravenous amino acid + CE vs. standard nutrition	Yes	EN ± PN + IV AA	72–96 h after ICU admission	5–10d	P:1.3 + (0.25–0.4) g/kg/d E: IC-guided	P: 1.3 g/kg/d E: IC-guided	P: 1.57 ± 0.27 g/kg/d E: 25.2 ± 4.76 kcal/kg/d	P: 1.23 ± 0.18 g/kg/d E: 24.23 ± 4.33 kcal/kg/d	Primary: muscle CSA; Secondary: muscle quality, strength, and function
Verceles et al. (2023) (51)	USA	S	16/23	Critically ill patients	*NMES+HPRO+PT vs. Standardized	Yes	EN or PN	Along with ICU care	14d	P: 1.4 ± 0.2 g/kg/d E: Penn State equation;22.7 ± 4.1 kcal/kg/d	P: 1.3 ± 0.2 g/kg/d E: Penn State equation;21.5 ± 4.8 kcal/kg/d	P: 1.3 ± 0.4 g /kg/d E: 20.1 ± 7.5 kcal/kg/d	P: 0.8 ± 0.4 g/kg/d E: 15.3 ± 6.8 kcal/kg/d	Primary: lower extremity muscle volume and CSA, nitrogen balance, delirium, MVD
Wang et al. (2024) (52)	China	S	86/87	Critically ill patients	High protein vs. Low protein	No	EN	NA	NA	P: 1–4d 1.5 g/kg/d E: 1-3d 20 kcal/kg/d, 4d 25 kcal/kg/d	P: 1–3d 0.8 g/kg/d; 4d:1.5 g/kg/d E: same	NA	NA	Primary: 28-day mortality Secondary: MVD, ICU discharge, adverse events, biochemical indicators, rectus femoris CSA
Zhang et al. (2022) (53)	China	S	20/21	PMV patients	Intensive nutrition vs. Standard nutrition	No	EN	Within 24–48 h	ICU stay	P: 2.0 g/kg/d E: 25–30 kcal/kg/d	P: 1.2 g/kg/d E: 25–30 kcal/kg/d	P: 1.70 ± 0.21 g/kg/d E: 33.5 ± 2.8 kcal/kg/d	P: 1.06 ± 0.21 g/kg/d E: 25.8 ± 4.8 kcal/kg/d	Primary: diaphragm atrophy, muscle mass Secondary: respiratory mechanics indices, ICU/hospital stay, ICU mortality

*, Interventions with rehabilitation, a total of 8 studies; S, single; M, multi; NA, not available; RCT, randomized controlled trial; EN, enteral nutrition; PN, parenteral nutrition; PCS, physical component summary score; MVD, mechanical ventilation days; ICU, intensive care unit; REE, Resting Energy Expenditure; NMES, Neuromuscular electrical stimulation; HPRO, high protein supplementation; HRQoL, health related quality of life; TBI: traumatic brain injury; WBC, white blood cell; APACHE II, acute physiologic and chronic health evaluation; NUTRIC, nutrition risk in the critically ill score; QMLT, quadriceps muscle layer thickness; CE, cycle ergometry; EMS, muscle electrical stimulation; PIICS, persistent inflammation, immunosuppression, and catabolism syndrome; CSA, cross-sectional area; SOFA, sequential organ failure assessment; ARF, acute renal failure; IBW, ideal body weight; PT, physical treatment; PMV, prolonged mechanical ventilation; IC-guided, guided by indirect calorimetry; BUN, Blood Urea Nitrogen; MAC, mid-arm circumference; MUAC, Mid-Upper Arm Circumference. ASPEN, American Society for Parenteral and Enteral Nutrition.

EN±PN indicates that EN was the primary route, with PN provided when nutritional targets could not be achieved.

EN or PN indicates that either EN or PN was selected according to clinical condition or physician judgment.

EN/PN + IV AA indicates that baseline nutrition was provided through EN or PN, with additional intravenous amino acids used in the higher-protein group.

EN±PN + IV AA indicates that EN was the primary route, PN was provided when required, and intravenous amino acids were additionally used to achieve the higher protein target.

Regarding population coverage, 30 RCTs (11–13, 24–32, 34–37, 39–46, 48–53) focused on the general critically ill population. Three RCTs (33, 38, 47) included patients with AKI. Eight RCTs combined protein interventions with physical rehabilitation (26, 27, 39, 40, 42, 44, 50, 51) including neuromuscular electrical stimulation (NMES), early exercise, cycle ergometry, muscle electrical stimulation, physical exercise, and NMES combined with physical treatment (Table 1; Supplementary Table S7).

### 3.3 Nutritional delivery

The details regarding nutritional delivery for the higher- and lower-protein groups are provided in Table 1 and Supplementary Table S8. Interventions were initiated within 1–2 days of admission in 22 RCTs (11, 13, 24, 25, 27, 28, 30–33, 35–37, 39, 41, 42, 44–46, 49, 51, 53); 2 RCT began within 3 days (26, 43), three within 4 days (38, 48, 50), and one after 10 days (34); 5 RCTs did not specify initiation timing (12, 29, 40, 47, 52). The duration of nutrition delivery ranged from 3 to 28 days. Four RCTs (12, 43, 48, 53) only noted that the delivery period aligned with the ICU stay. Regarding the route of nutrition delivery, 25 RCTs primarily used enteral nutrition (EN) (12, 13, 24–28, 30, 31, 34, 35, 37, 39–46, 48–50, 52, 53), of which 9 permitted supplemental PN when required (12, 25, 26, 28, 34, 42, 44, 49, 50); 2 used exclusive parenteral nutrition (PN) (36, 47); 6 allowed either EN or PN according to the patient’s clinical condition (11, 29, 32, 33, 38, 51).

Among the 33 included RCTs, 23 (11, 12, 24–29, 34–38, 40–42, 44, 45, 48, 50–53) provided numerical prescribed protein targets in g/kg/day for both groups, and 3 studies reported targets for the higher-protein group only. The remaining studies reported protein doses in alternative units (e.g., g/day, g/L, or percentage of energy), which were not converted to g/kg/day. Among the 23 studies with complete bilateral data, the sample-size-weighted mean prescribed protein target was 1.69 g/kg/day in the higher protein groups and 0.75 g/kg/day in the lower protein groups. Regarding energy study design, of the 33 included RCTs, 27 studies were classified as isocaloric by design, using the same method and magnitude of energy targeting, whereas the remaining 6 studies (11, 25, 32, 41, 43, 46) were classified as non-isocaloric by design. Actual protein and energy delivery was also extracted and reported separately, details of nutrition delivery as shown in Table 1 and Supplementary Table S8.

### 3.4 Literature quality and bias assessment

The results of the risk of bias assessment are presented in Supplementary Figure S1. Overall, 9 RCTs (24, 26, 30, 35, 41, 43, 44, 47, 53) had a high risk of bias, 13 (11, 25, 27, 29, 32–34, 40, 42, 46, 48, 50, 51) showed some concern, and 11 (12, 13, 28, 31, 36–39, 45, 49, 52) had low risk of bias. Regarding blinding methods, 9 RCTs (12, 28, 31, 35, 36, 40, 45, 46, 49) employed double-blind designs, 9 (26, 27, 29, 34, 37, 38, 48, 51, 52) used single-blind designs, 13 (11, 13, 24, 30, 33, 39, 41–44, 47, 50, 53) were open-label, and 2 (25, 32) did not report blinding information. In terms of missing data, 6 RCTs (27, 32, 43, 45, 47, 48) reported no dropouts and 14 (12, 13, 25, 28, 31, 33, 36–39, 49, 50, 52, 53) applied intention-to-treat (ITT) analyses; the remaining 13 (11, 24, 26, 29, 30, 34, 35, 40–42, 44, 46, 51) did not use ITT analysis.

### 3.5 Clinical outcomes

#### 3.5.1 Mortality

Mortality was reported in 26 RCTs (11–13, 24–28, 30–34, 36–39, 41–44, 47–49, 52, 53) (*n* = 11,099). No significant difference in all-cause mortality was observed between the higher- and lower-protein groups (RR 1.02, 95% CI 0.97 to 1.08) (Figure 2A); in TSA, the Z-curve reached the optimal information size and crossed the futility boundary (Figure 2B). No differences in 30-day (11 RCTs, RR1.04, 95% CI 0.96 to 1.13), 60-day (4 RCTs, RR 1.07, 95% CI 0.96 to 1.20), 90-day (6 RCTs, RR 1.04, 95% CI 0.98 to 1.11), 180-day (2 RCTs, RR 0.97, 95% CI 0.36 to 2.62), ICU (11 RCTs, RR 1.04, 95% CI 0.95 to 1.14), or in-hospital mortality (11 RCTs, RR 1.03, 95% CI 0.95 to 1.12) were observed between the groups (Supplementary Figure S3; Supplementary Table S6). In the group of patients receiving physical rehabilitation, higher protein delivery was associated with a reduction in mortality (RR 0.68, 95% CI 0.50 to 0.93) (Figure 2C); however, among patients with AKI, higher protein intake was associated with a significant increase in mortality (RR 1.46, 95% CI 1.14 to 1.87) (Figure 2D).

**Figure 2:**
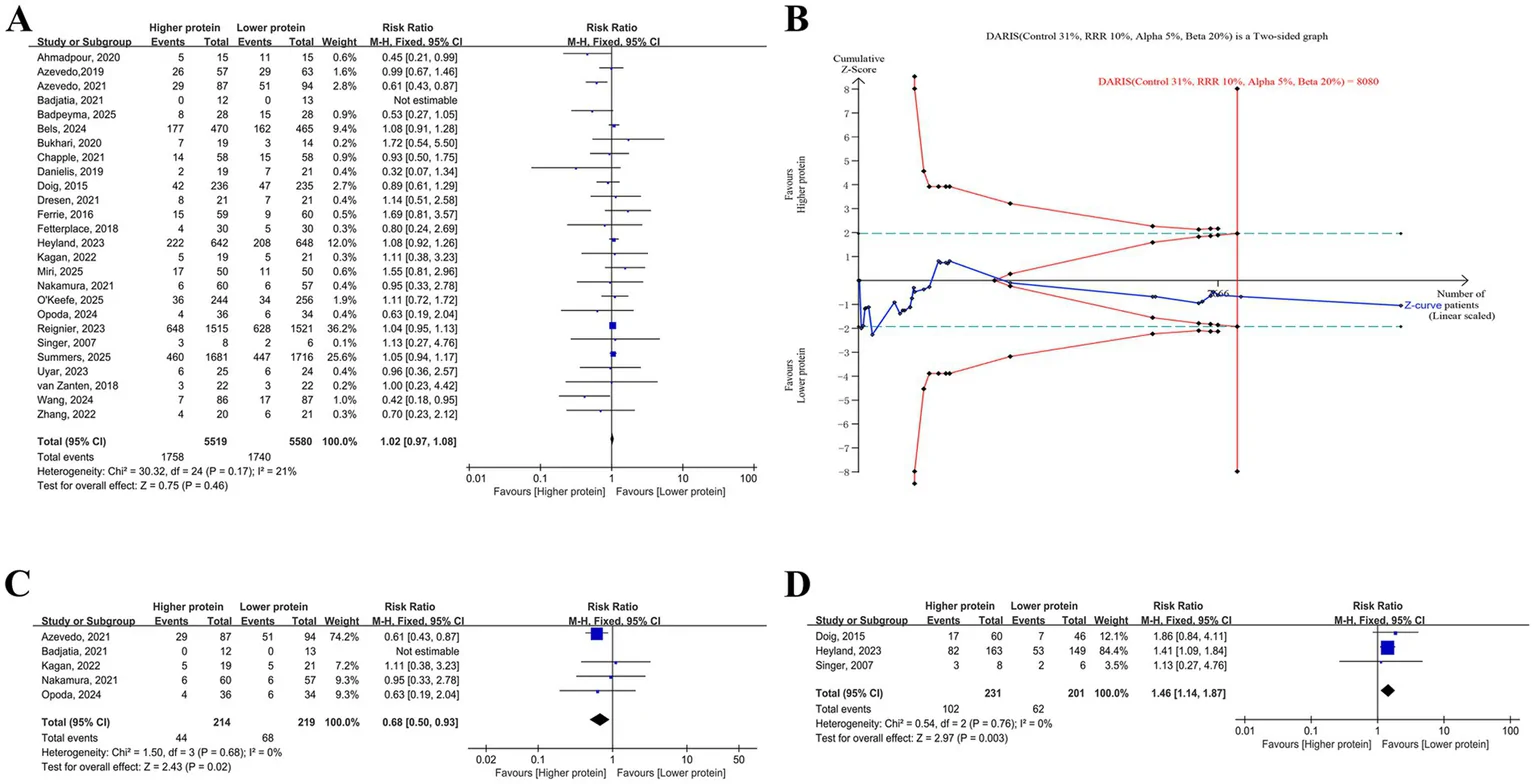
Mortality and trial sequential analysis (TSA). **(A)** All-cause mortality. **(B)** TSA of mortality. **(C)** Mortality of higher protein combining with rehabilitation. **(D)**. Mortality of AKI population. DARIS, diversity adjusted required information size is the calculated optimum sample size for statistical inference; RRR, relative risk reduction.

#### 3.5.2 MV duration and ICU length of stay

MV duration was reported in 21 RCTs (11–13, 25, 26, 28, 32, 34–39, 42, 44–46, 49, 51–53) (*n* = 9,992), with no significant difference between the two groups (MD −0.48, 95% CI −1.08 to 0.13). Subgroup analyses (26, 39, 42, 44, 51) revealed reduced MV duration in the higher-protein combined with physical rehabilitation group (MD −1.45, 95% CI −2.61 to −0.29). ICU LOS was assessed in 24 RCTs (11–13, 24–28, 30, 31, 33–39, 42, 45, 46, 49, 51–53) (*n* = 10,564), with no between-group difference (MD −0.18, 95% CI −0.86 to 0.51), consistent across subgroups. In TSA, the Z-curve crossed the futility boundary for both MV duration and ICU LOS, with MV duration reaching the optimal information size (Figure 3; Supplementary Figures S4, S5).

**Figure 3:**
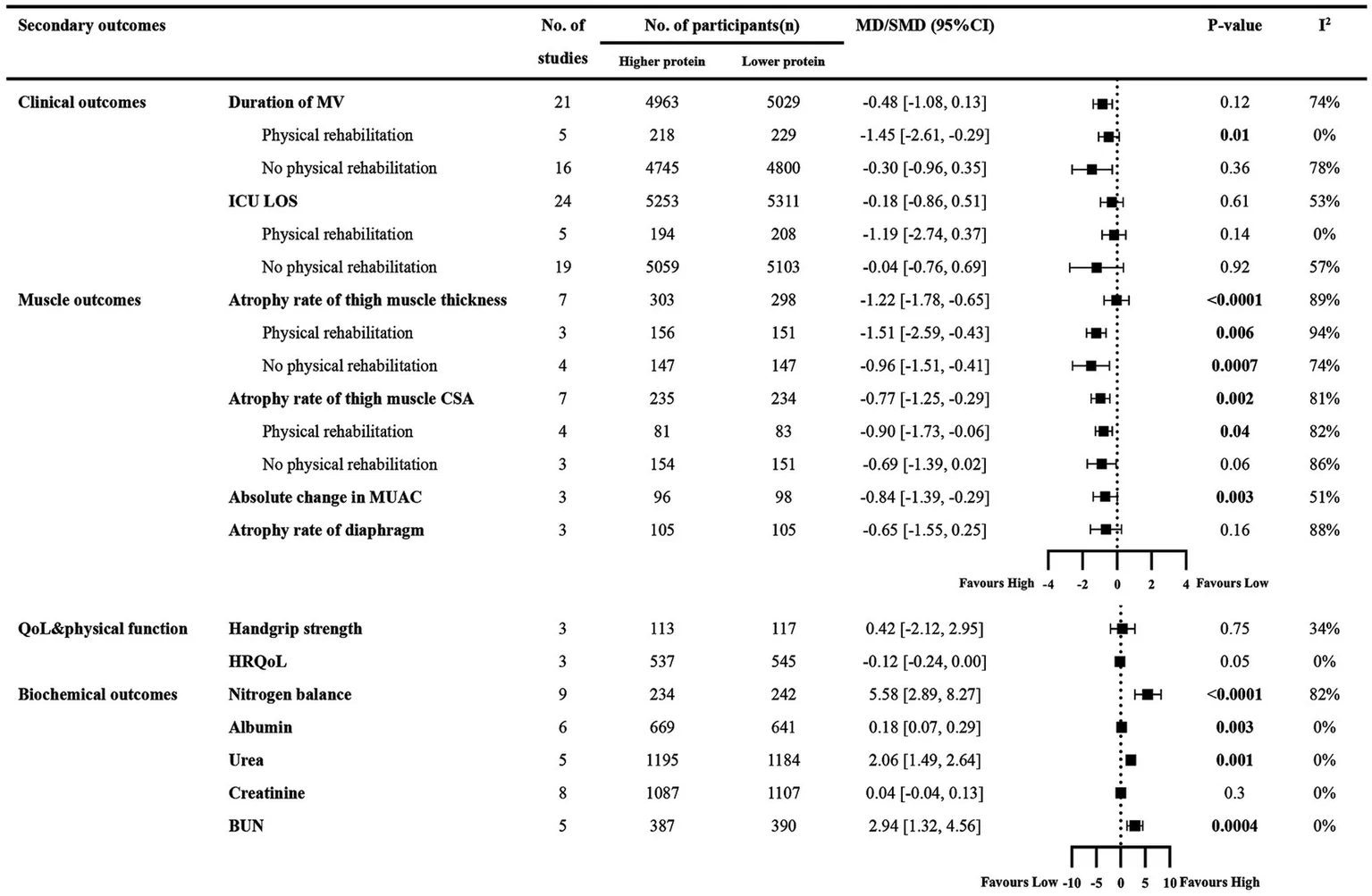
Secondary outcomes of the included RCTs. MV, mechanical ventilation; ICU, intensive care unit; LOS, length of stay; CSA, cross sectional area; MUAC, mid-upper arm circumference; QoL, quality of life; HRQoL, health-related quality of life; BUN, blood urea nitrogen.

#### 3.5.3 Rate of muscle atrophy, handgrip strength, and HRQoL

Our meta-analyses revealed that higher protein delivery significantly reduced the rate of thigh muscle atrophy in terms of both thickness (34, 40, 42, 44, 45, 48, 52) (SMD −1.22, 95% CI −1.78 to −0.65) and CSA (27, 35, 44, 45, 50–52) (SMD −0.77, 95% CI −1.25 to −0.29). Subgroup analyses also revealed a reduction in the rate of thigh muscle atrophy in the higher-protein combined with physical rehabilitation group. In the higher protein group, a significant reduction in the atrophy rate of MUAC was observed (MD −0.84, 95% CI −1.39 to −0.29) (28, 37, 41). However, no significant differences were observed in handgrip strength (MD 0.42, 95% CI −2.12 to 2.95) (25, 36, 37), or HRQoL measurements (SMD −0.12, 95% CI −0.24 to 0.00) (12, 31, 42) (Figure 3; Supplementary Figures S6, S7).

#### 3.5.4 Biochemical results

Our meta-analyses showed that higher protein delivery significantly increased nitrogen balance (24, 27, 29, 32, 36, 41, 47, 51, 53) (MD 5.58, 95% CI 2.89 to 8.27), albumin levels (13, 28, 35, 37, 40, 42) (SMD 0.18, 95% CI 0.07 to 0.29), urea concentrations (13, 28, 31, 38, 50) (MD 2.06, 95% CI 1.49 to 2.64), and BUN levels (35, 42, 43, 47, 49) (MD 2.94, 95% CI 1.32 to 4.56). However, no significant differences were observed in creatinine levels (28, 31, 32, 38, 42, 43, 47, 50) between the groups (Figure 3; Supplementary Figure S8).

#### 3.5.5 Adverse events

In terms of adverse events, our meta-analyses indicated that higher protein delivery was significantly associated with an increased risk of diarrhea (11, 31, 37, 42, 43, 49) (RR 1.15 95% CI 1.05 to 1.25), regurgitation/vomiting (11, 31, 42, 43, 49) (RR 1.22, 95% CI 1.08 to 1.38), and receipt of promotility agents (11, 12, 31) (RR 1.48, 95% CI 1.31 to 1.68). No significant differences were found between the two groups for feeding intolerance (12, 28, 37, 49), constipation (11, 49), receipt of RRT/incident RRT (11–13, 31, 33, 34, 38, 43), and infection in the ICU (11, 12, 27, 34, 42–44) (Figure 4; Supplementary Figure S9).

**Figure 4:**
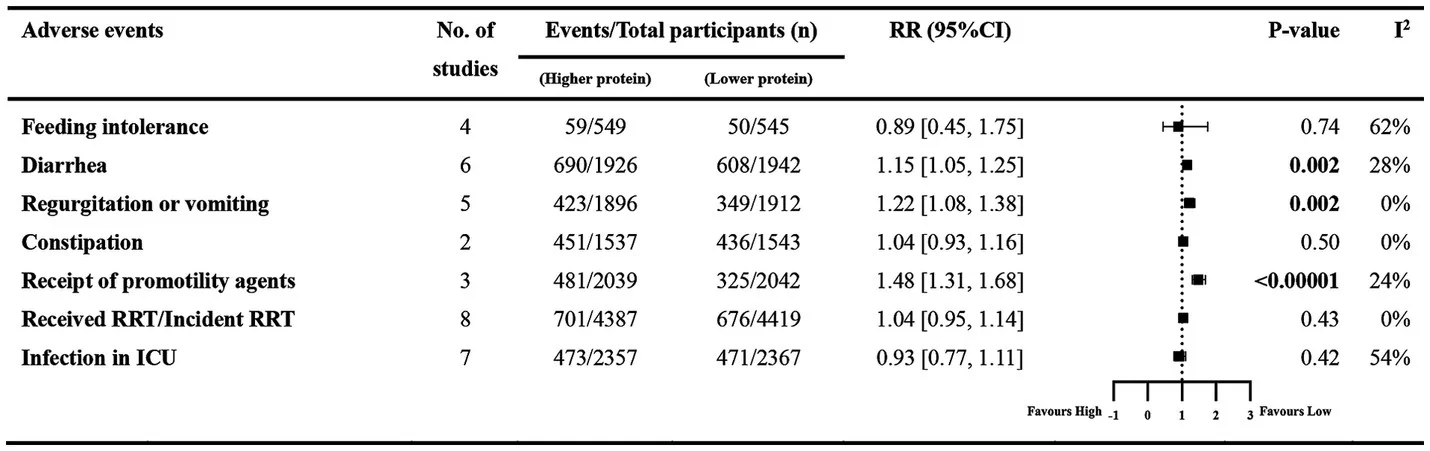
Adverse events. RRT, renal-replacement therapy; ICU, intensive care unit.

### 3.6 Sensitivity analysis

We further performed sensitivity analyses of all-cause mortality using 1.2, 1.3 and 1.5 g/kg/day as alternative cut-offs to distinguish the higher- and lower-protein groups. No significant difference was observed at either the 1.2 g/kg/day cut-off (10 RCTs; RR 1.00, 95% CI 0.87 to 1.15),1.3 g/kg/day cut-off (11 RCTs; RR 1.03, 95% CI 0.93 to 1.15) or the 1.5 g/kg/day cut-off (15 RCTs; RR 0.98, 95% CI 0.89 to 1.08), consistent with the primary analysis (Supplementary Table S9).

Sensitivity analyses restricted to studies with the same prescribed energy target in both groups were conducted. No significant difference in all-cause mortality was observed among these isocaloric-design RCTs (21 RCTs; RR 1.01, 95% CI 0.94 to 1.08) (Supplementary Table S9). For most secondary outcomes, the results were consistent with the primary analysis. However, the statistical significance changed for diarrhea (4 RCTs; RR 0.91, 95% CI 0.71 to 1.17) and regurgitation or vomiting (3 RCTs; RR 0.90, 95% CI 0.51 to 1.59), neither of which showed a statistically significant between-group difference (Supplementary Figure S10).

### 3.7 Quality of evidence and publication bias

The certainty of evidence, as assessed using the GRADE approach, varied across outcomes (Supplementary Figure S11). High certainty: HRQoL, thigh muscle CSA atrophy rate, and urea; Moderate certainty: mortality, MV duration, handgrip strength, albumin, creatinine, BUN levels, constipation, use of promotility agents, and receipt of RRT or incident RRT; Low certainty: ICU LOS, thigh muscle thickness atrophy rate, absolute change in MUAC, nitrogen balance, feeding intolerance, diarrhea, and regurgitation/vomiting; Very low certainty was assigned to diaphragm atrophy rate, and ICU-acquired infection.

Funnel plot asymmetry was assessed using Egger’s and Begg’s tests with at least 10 contributing studies. No clear evidence of publication bias was observed for mortality (Egger’s *p* = 0.45; Begg’s *p* = 0.52). For MV duration and ICU length of stay, Egger’s tests indicated possible funnel plot asymmetry (*p* = 0.03 and *p* = 0.04, respectively), whereas Begg’s tests were not statistically significant (*p* = 0.12 and *p* = 0.18, respectively). The trim-and-fill analyses did not materially alter the overall conclusions (Supplementary Table S10).

## 4 Discussion

This systematic review and meta-analysis demonstrated that higher versus lower protein intake did not significantly improve clinical outcomes, including all-cause mortality across multiple time points ICU length of stay, or MV duration, with TSA confirming these null findings. Although higher protein intake was associated with beneficial effects on muscle outcomes (atrophy rate of thigh muscle thickness and CSA), no significant differences were observed for diaphragm atrophy or handgrip strength. Higher protein also improved nitrogen balance, and elevated serum albumin levels, but was linked to elevated urea and BUN concentrations and a trend toward worse HRQoL. Subgroup analyses revealed that higher protein intake was linked to increased mortality in patients with AKI. However, in the pre-specified subgroup of patients receiving physical rehabilitation, higher protein delivery was associated with reduced mortality, shorter MV duration, and benefits effects on muscle outcomes.

Consistent with a prior meta-analysis (10) of 23 RCTs (*n* = 3,303) and recent large enteral nutrition trials (11, 13, 38), our findings demonstrate that higher versus lower protein delivery does not reduce all-cause mortality across multiple time points. TSA confirmed this neutral finding. Nevertheless, substantial clinical heterogeneity warrants cautious interpretation. Included studies spanned the acute phase of critical illness across diverse ICU populations and underlying conditions, with considerable variation in protein definitions, energy comparability, and delivery routes. Regarding protein definitions, consistent with the approach taken by the prior meta-analysis (10), which also adopted study-specific definitions for higher- and lower-protein groups, we defined groups according to each trial’s original allocation rather than imposing a fixed cut-off. This approach was justified by the lack of consensus on optimal protein targets and the marked variability across recent landmark RCTs (e.g., 2.0 vs. 1.3 g/kg/day; 1.0–1.3 vs. 0.2–0.4 g/kg/d; ≥2.2 vs. ≤ 1.2 g/kg/d), which together contributed over 8,000 participants (11–13, 38). Therefore, we performed sensitivity analyses using conventional cut-offs of 1.2, 1.3, and 1.5 g/kg/day, all of which yielded directionally consistent, non-significant results. To address energy heterogeneity, we additionally restricted the analysis to isocaloric-design RCTs, which produced a similar null result. However, the limited number of studies using exclusive PN or post-pyloric feeding precluded subgroup analysis by delivery modality. Additionally, the low statistical power for mortality as a secondary outcome in most included RCTs may limit detection of subtle effects. In summary, within the range of analytical frameworks explored, higher protein delivery did not confer a mortality advantage over lower delivery, although residual confounding from unmeasured clinical or nutritional variables cannot be excluded.

Research on nutritional and physical rehabilitation interventions for functional recovery during critical illness still limited. A previous systematic review (10) of only two small studies suggested a trend toward improved self-reported physical function with combined higher protein intake and early rehabilitation. Our subgroup analyses further demonstrated that higher protein intake combined with physical rehabilitation significantly reduced mortality, MV duration and muscle atrophy in critically ill patients. Biologically, this is plausible: our analysis showed that higher protein alone improved nitrogen balance, serum albumin, and attenuated muscle atrophy (mainly via ultrasound). But a recent isotopic tracer RCT (56) provides a mechanistic explanation that critically ill patients exhibit severe anabolic resistance, whereby increasing protein intake elevates plasma amino acids without stimulating muscle protein synthesis. Importantly, while higher protein alone improved these surrogates without reducing mortality, adding rehabilitation was linked to survival and clinical benefits, suggesting a synergistic interaction between nutrition and exercise. Previous studies have proposed that exercise can counteract critical illness-induced anabolic resistance, reduce complications, and enhance activity levels (57, 58). However, our findings are derived solely from small-sample studies and subgroup analyses. Therefore, multicentre, sufficiently powered RCTs with a two-by-two factorial design that compares higher versus lower protein intake combined with rehabilitation versus no rehabilitation are needed. Besides, additional studies are also warranted to explore the mechanisms and influence of higher protein intake and physical therapy on muscle anabolism and protein utilization.

Pre-specified subgroup analyses revealed that higher protein intake was associated with increased mortality in patients with AKI at ICU admission, consistent with prior evidence. This is not contradictory to the neutral RRT findings in the overall cohort: higher protein did not trigger *de novo* dialysis at the population level, but could potentially adversely affect patients with established renal failure. Despite varying AKI definitions across the three included studies, the directional consistency of these findings underscores their robustness. A isotopic evidence (59) shows profound anabolic resistance in critical illness, with muscle protein synthesis ~60% lower than in healthy controls. Together with our finding of increased urea levels, this suggests surplus protein is converted to urea rather than muscle. Prior studies (2, 60) indicates excess protein exacerbates ureagenesis and accumulates toxic metabolites, amplifying renal burden and mortality risk. While absolute urea increases were modest, their relevance in AKI is unclear. Given the limited studies, both the protein–AKI mortality association and its mechanisms require further investigation.

Our meta-analysis of three studies identified a trend toward worse HRQoL with higher protein intake(*p* = 0.05). Among the included studies, PRECISe (*n* = 935) (12) demonstrated that high enteral protein provision resulted in statistically worse as its primary endpoint, employing a longitudinal analysis across three timepoints that included non-survivors (assigned a score of zero). Similarly, Chapple et al. (31) (*n* = 116) showed a consistent trend that lower protein intake was associated with better EQ-5D-5L health utility scores at 90 days, whereas Nakamura et al. (42) (*n* = 117) reported no between-group differences. Mechanistically, excessive amino acid supply may exceed the body’s protein synthetic capacity, driving increased amino acid oxidation and accumulation of potentially toxic deamination byproducts (61). Consistent with this hypothesis, our results demonstrate that higher protein delivery elevated blood urea and BUN levels, which may link excess protein to adverse effects on clinical outcomes and quality of life. In summary, the mechanism underlying the trend toward deleterious effects of higher protein intake remain unknown, warranting further investigation.

Our analysis also revealed a significantly higher incidence of adverse events among critically ill patients received higher protein, including diarrhea, regurgitation, vomiting, and promotility agent use. However, our sensitivity analysis with isocaloric design showed that diarrhea and vomiting were no longer significantly associated with higher protein provision. This indicates that the apparent gastrointestinal intolerance was driven primarily by concurrent caloric delivery or greater feeding volumes rather than protein itself, aligning with a recent rigorous systematic review and meta-analysis (62) demonstrating that higher enteral protein alone does not increase diarrhea risk. Moreover, included trials enrolled acute-phase critically ill patients with varying etiologies and disease severities, delivering protein via enteral (post-pyloric feeding data uncertain) and parenteral routes, introducing additional confounding. Future research should disentangle how protein dosing, energy delivery, feeding routes, and feeding volumes collectively influence gastrointestinal tolerance in acute critical illness, alongside individual factors.

Our findings have important implications for both clinical practice and future research. In clinical practice, they prompt a recalibration of expectations regarding high-dose protein in critically ill patients. The divergent outcomes across subgroups, particularly the potential harm observed in patients with AKI, highlight the urgent need for risk-stratified prescribing strategies. In parallel, the signal of benefit when higher protein is combined with physical rehabilitation suggests that nutrition should be an integral component of a multimodal recovery strategy. Additionally, heterogeneity in energy comparability, variability in protein definitions, and the paucity of studies using post-pyloric feeding or exclusive parenteral nutrition limit the generalizability of our findings. Future trials should therefore prioritize mechanism-driven, stratified designs that simultaneously account for renal function, delivery route, and functional rehabilitation, rather than treating critical illness as a homogeneous entity. Large-scale factorial trials are also needed not only to confirm the synergistic benefits of protein plus exercise, but also to dissect the underlying mechanisms of this interaction.

There were also several limitations to this study. First, significant heterogeneity in energy and protein codelivery across the included studies, along with inconsistent control and reporting of total energy intake, may have confounded the estimated effects of protein interventions. Second, variability in protein delivery strategies, including differences in dosage, timing, and administration routes, limits the generalizability of the findings and complicates direct clinical translation. Third, although pre-specified subgroup analyses were performed, limited reporting in the source studies prevented further stratification of high-risk populations (e.g., patients with specific organ dysfunctions or distinct nutritional statuses). In particular, the scarcity of data on post-pyloric feeding and exclusive parenteral nutrition prevented subgroup analysis by delivery modality. Finally, potential publication bias cannot be excluded, and methodological limitation (including incomplete reporting of analysis populations, adherence, and missing data, as well as unavoidable performance bias from unblinded nutritional interventions) may affect the certainty of the evidence.

## 5 Conclusion

In critically ill patients, the present meta-analysis revealed that higher protein delivery does not improve survival, shorten MV duration, or reduce ICU length of stay, even after accounting for energy balance and applying alternative protein cut-offs. While higher protein attenuates muscle wasting and supports nutritional status, it is associated with a trend toward worse health-related quality of life. Gastrointestinal intolerance appears driven by feeding volume rather than protein itself, and higher protein may increase mortality in patients with AKI. Notably, combining higher protein with physical rehabilitation yields survival and clinical benefits. Future trials should employ factorial designs to confirm this synergy and explore the underlying mechanisms.

## Data Availability

The original contributions presented in the study are included in the article/Supplementary material, further inquiries can be directed to the corresponding author.
