# Supplementary material for: Effects of higher versus lower protein intake in critically ill patients: a systematic review and meta-analysis
Source: Front Nutr. 2026 Sep 25;13:1900749. doi: 10.3389/fnut.2026.1900749 (PMC13649726; doi:10.3389/fnut.2026.1900749)
Supplement: Supplementary file 1 [file Supplementary_file_1.docx]

**Effects of higher versus lower protein intake in critically ill patients: A systematic review and meta-analysis**

| Results |  |
| --- | --- |
| 1. Table S2. Search strategy and supplementary methods | P 2-3 |
| 2. Table S3. Inclusion and exclusion criteria of the included RCTs | p 4-6 |
| 3. Table S4. Demographic details of the included RCTs | p 7-8 |
| 4. Table S5. Admission reason of the included RCTs | p 9-10 |
| 5. Table S6. Reported and selected outcome time points | P 11 |
| 6. Table S7. Details of physical rehabilitation | P 12-13 |
| 7. Table S8. Details of nutrition delivery | p 14-17 |
| 8.Table S9. Sensitivity analysis of mortality | P 18-19 |
| 9. Table S10 Publication bias for 4 risk factors | P 20 |
| 10. Fig S1. Assessment on risk of bias for included RCTs | p 21 |
| 11. Fig S2. Funnel plot for included studies | p 22 |
| 12. Fig S3. 30-, 60-, 90-, 180-day, ICU, Hospital Mortality analysis for included RCTs | p 23 |
| 13. Fig S4. ICU length of stay (LOS) and subgroup analysis for included RCTs | p 24 |
| 14. Fig S5. Duration of MV and subgroup analysis for included RCTs | p 25 |
| 15. Fig S6. Atrophy rate of thigh muscle thickness, thigh muscle cross sectional area (CSA), and diaphragm analysis for included RCTs | p 26 |
| 16. Fig S7. Handgrip strength and HRQoL analysis for included RCTs | p 27 |
| 17. Fig S8. Biochemical tests analysis for included RCTs | p 28 |
| 18. Fig S9. Adverse effects analysis for included RCTs | p 29 |
| 19. Fig S10. Sensitivity analysis: studies with the same prescribed energy target in both groups. | P 30-37 |
| 20. Fig S11. The GRADE assessment of the certainty of the evidence | p 38-39 |

Table S2. Search strategy and supplementary methods

**Search strategy**

A) PubMed

| # | Query | Results from 28 July 2026 |
| --- | --- | --- |
| 1 | (("Critical Care"[MeSH Terms] OR ("care critical"[Title/Abstract] OR "intensive care"[Title/Abstract] OR "care intensive"[Title/Abstract] OR "critically ill"[Title/Abstract] OR "critical illness"[Title/Abstract] OR "mechanical ventilation"[Title/Abstract] OR "Ventilated"[Title/Abstract] OR "ICU"[Title/Abstract] OR "acute lung injury"[Title/Abstract] OR "multiple organ dysfunction syndrome"[Title/Abstract])) AND ("Nutrition Therapy"[MeSH Terms] OR ("therapy nutrition"[Title/Abstract] OR "Nutrition"[Title/Abstract] OR "Nutritional"[Title/Abstract] OR "Caloric"[Title/Abstract] OR "Calorie"[Title/Abstract] OR "Protein"[Title/Abstract] OR "Energy"[Title/Abstract] OR "Feeding"[Title/Abstract] OR "amino acids"[Title/Abstract]))) AND ((randomizedcontrolledtrial[Filter]) AND (humans[Filter]) AND (alladult[Filter] OR youngadult[Filter] OR adult[Filter] OR middleagedaged[Filter] OR middleaged[Filter] OR aged[Filter] OR 80andover[Filter])) | 1909 |

B) Embase

| # | Query | Results from 28 July 2026 |
| --- | --- | --- |
| 1 | 'intensive care'/exp OR 'care, intensive':ti,ab OR 'intensive therapy':ti,ab OR 'intensive care':ti,ab OR 'therapy, intensive':ti,ab OR 'critically ill':ti,ab OR 'critical illness':ti,ab OR 'mechanical ventilation':ti,ab OR 'ventilated':ti,ab OR 'icu':ti,ab OR 'acute lung injury':ti,ab OR 'multiple organ dysfunction syndrome':ti,ab | 1459431 |
| 2 | 'diet therapy'/exp OR 'diet intervention':ti,ab OR 'diet treatment':ti,ab OR 'dietary intervention':ti,ab OR 'dietary therapy':ti,ab OR 'dietary treatment':ti,ab OR 'diet therapy':ti,ab OR 'nutrition therapy':ti,ab OR 'nutritional therapy':ti,ab OR 'nutrition':ti,ab OR 'nutritional':ti,ab OR 'caloric':ti,ab OR 'calorie':ti,ab OR 'protein':ti,ab OR 'energy':ti,ab OR 'feeding':ti,ab OR 'amino acids':ti,ab | 6172021 |
| 3 | #1 AND #2 AND 'article'/it AND [humans]/lim AND [randomized controlled trial]/lim | 6668 |
| 4 | #3 AND ('adult':ag OR 'aged':ag OR 'middle aged':ag OR 'very elderly':ag OR 'young adult':ag) AND [embase]/lim | 4704 |

C) Web of Science

| # | Query | Results from 28 July 2026 |
| --- | --- | --- |
| 1 | TS=("critical care" OR "intensive care" OR ICU OR "critically ill"OR "critical illness" OR "mechanical* ventilat*" OR "acute respiratory distress syndrome" OR ARDS OR "acute lung injur*" OR "multiple organ dysfunction") | 481157 |
| 2 | TS=((protein* OR "amino acid*") NEAR/10 (nutrition* OR feed* OR intake OR deliver* OR provision OR dose* OR target* OR supplement*)) | 420467 |
| 3 | TS=("randomized controlled trial*" OR "randomised controlled trial*" OR "controlled clinical trial*" OR (random* NEAR/3 (trial* OR allocat* OR assign* OR group*))) | 1115586 |
| 4 | #1 AND #2 AND #3 | 590 |

D) Cochrane

| # | Query | Results from 28 July 2026 |
| --- | --- | --- |
| 1 | MeSH descriptor: [Critical Care] explode all trees | 3126 |
| 2 | (Care, Critical):ti,ab,kw OR (Intensive Care):ti,ab,kw OR (Care, Intensive):ti,ab,kw OR (Critically ill):ti,ab,kw OR (Critical illness):ti,ab,kw OR (Mechanical ventilation):ti,ab,kw OR (Ventilated):ti,ab,kw OR (ICU):ti,ab,kw OR (Acute lung injury):ti,ab,kw OR (multiple organ dysfunction syndrome):ti,ab,kw | 81192 |
| 3 | #1 OR #2 | 81201 |
| 4 | MeSH descriptor: [Nutrition Therapy] explode all trees | 13590 |
| 5 | (Therapy, Nutrition):ti,ab,kw OR (Nutrition):ti,ab,kw OR (Nutritional):ti,ab,kw OR (Caloric):ti,ab,kw OR (Calorie):ti,ab,kw OR (Protein):ti,ab,kw OR (Energy):ti,ab,kw OR (Feeding):ti,ab,kw OR (Amino Acids):ti,ab,kw | 222301 |
| 6 | #4 OR #5 | 225396 |
| 7 | (Randomized controlled trial):ti,ab,kw OR (Randomized):ti,ab,kw OR (Randomised):ti,ab,kw OR (Random):ti,ab,kw | 1367547 |
| 8 | #3 AND #6 AND #7 | 7909 |

E) Scopus

| # | Query | Results from 28 July 2026 |
| --- | --- | --- |
| 1 | ( ( TITLE-ABS-KEY ( critical AND care ) OR TITLE-ABS-KEY ( care , AND critical ) OR TITLE-ABS-KEY ( intensive AND care ) OR TITLE-ABS-KEY ( care , AND intensive ) OR TITLE-ABS-KEY ( critically AND ill ) OR TITLE-ABS-KEY ( critical AND illness ) OR TITLE-ABS-KEY ( mechanical AND ventilation ) OR TITLE-ABS-KEY ( ventilated ) OR TITLE-ABS-KEY ( icu ) OR TITLE-ABS-KEY ( acute AND lung AND injury ) OR TITLE-ABS-KEY ( multiple AND organ AND dysfunction AND syndrome ) ) ) AND ( ( TITLE-ABS-KEY ( nutrition AND therapy ) OR TITLE-ABS-KEY ( therapy , AND nutrition ) OR TITLE-ABS-KEY ( nutrition ) OR TITLE-ABS-KEY ( nutritional ) OR TITLE-ABS-KEY ( caloric ) OR TITLE-ABS-KEY ( calorie ) OR TITLE-ABS-KEY ( protein ) OR TITLE-ABS-KEY ( energy ) OR TITLE-ABS-KEY ( feeding ) OR TITLE-ABS-KEY ( amino AND acids ) ) ) AND ( TITLE-ABS-KEY ( randomized AND controlled AND trial ) ) AND ( LIMIT-TO ( DOCTYPE , "ar" ) ) | 7515 |

Supplementary Methods- Trial Sequential Analysis

TSA aimed for a 5% risk for type I error (Alpha) and a 20% risk for type II error (Beta, power 80%) in this study. Fixed-effects model was used for I² < 50%; random-effects (Sidik - Jonkman, SJ) model for I² > 50%. Between-study heterogeneity was adjusted via diversity-estimate (D^2^). For categorical outcomes (mortality, adverse events), a 0.5 continuity correction was added for zero events. The effect size was set at 10%, based on a prior meta-analysis (https://doi.org/10.1186/s13054-023-04783-1) or clinically meaningful, realistic magnitude. The proportion of events in the control group was based on pooled observed rate from updated meta-analysis. For continuous outcomes, minimal important difference (MID) was based on patient/clinician-meaningful change (1 day for ICU length of stay and duration of mechanical ventilation). The variance was based on pooled observed standard deviation (SD) from current updated meta-analysis. If the cumulative Z-curve surpasses the trial sequential monitoring boundary/ futility boundary or reaches the Required Information Size (RIS), the evidence is sufficiently strong. If the Z-curve does not cross any boundaries and the RIS is not reached, it indicates insufficient evidence for a conclusion, necessitating more studies.

* This study selected the SJ model for TSA, as it enables a more refined estimation of between study heterogeneity variance to capture complex heterogeneity structures (e.g., coexistence of large - sample high - quality RCTs and small - sample studies), in line with the methodological rationale in the TSA manual.

| Table S3. Inclusion and exclusion criteria of the included RCTs | | |
| --- | --- | --- |
| Author, year | Inclusion criteria | Exclusion criteria |
| Ahmadpour, 2020 | The neurocritical patients (older than 16 years) with an estimated ICU stayed for more than five days and they were able to tolerate the enteral tube feeding included in the study. | The exclusion criteria were death during the first five days, encephalopathy, pregnancy, metabolic disorders, acute and chronic kidney failure, and proteinuria. |
| Azevedo, 2019 | Included were patients over 18 years of age, nonpregnant, submitted to mechanical ventilation, expectation of stay in the ICU was greater than 2 days, and admitted to the ICU from June 2016 to November 2017. | Patients were excluded if they were not expected to remain in the ICU for at least 3 days, had high-output bronchopleural fistulas, required an inspired fraction of oxygen (FiO2 )≥ 0.6, and presented evidence of severe cognitive dysfunction, which was identified through family information and patient evaluation performed by a hospital psychologist upon admission to the ICU. |
| Azevedo, 2021 | The trial only included patients aged above 18 years, who were admitted to ICU for more than 3 days, between June 2018 to June 2020, and were submitted to mechanical ventilation. | Patients were excluded for the following reasons: if they were pregnant, moribund, under ventilation for more than 96h before enrolment, and unable to walk without assistance before the acute illness that led to ICU admission (use of an assistance device to walk was not an exclusion criteria); if they suffered from severe cognitive impairment before hospitalization, neuromuscular diseases that compromised weaning from ventilation, acute pelvic fracture, unstable spinal trauma, and severe liver disease; if it was impossible to start a diet according to the institutional protocols; and if they did not sign the written informed consent. |
| Badjatia, 2021 | (1) Aneurysmal subarachnoid hemorrhage (SAH); (2) Aneurysmal repair within 48 hours of ictus; (3) Age >18 years; (4) Expected Neurological Care Unit stay >72 hours; (5) Hunt Hess grade≥2; (6) Modified Fisher score >1. | (1) Subarachnoid hemorrhage(SAH) from trauma or other non-aneurysmal causes; (2) Unlikely to survive 1 week post-hemorrhage; (3) ICU stay≤7 days; (4) BMI <15 or >40; (5) Allergy to whey protein; (6) Lower extremity paresis/spasticity within 48 hours; (7) Pre-morbid mRS >1; (8) Pregnancy, malignancy, etc.; (9) Ongoing seizures; (10) Prisoner. |
| Badpeyma, 2025 | Age between 18 and 80 years. Admission to the intensive care unit. Hemodynamic and metabolic stability within the first 24–48 hours. Requirement for enteral nutrition. Expected duration of invasive mechanical ventilation exceeding 72 hours. Written informed consent from the patient or legally authorized representative. | Inability to tolerate enteral nutrition because of anatomical or mechanical barriers, such as bowel obstruction. Exclusive indication for parenteral nutrition. Pregnancy or lactation. Current or previous acute or chronic renal failure. Hepatic encephalopathy. Receipt of the study intervention for fewer than four days. Inability to obtain written informed consent. Concurrent enrollment in another clinical trial. Frequent requirement for blood transfusions. Physician discretion that the patient was unsuitable for participation. |
| Bels, 2024 | Initiation of invasive mechanical ventilation within 24 h of ICU admission and an expected duration of invasive ventilation of 3 days or longer. | Exclusion criteria were contraindications for EN, moribund condition, BMI less than 18 kg/m², kidney failure with a no dialysis code, or hepatic encephalopathy. |
| Bibi,2025 | Critically ill adult patients of either gender who had been in ICUs, high-dependence units (HDUs) and step-down units (SDUs) for >48 hours and who were at nutritional risk, as assessed by NUTRIC score >3.12 | Patients under 18 years of age, those on physician-prescribed protein restrictions, or on ventilator support for >72 hours were excluded, and sp were patients with ICU stay <48 hours, and those expected to have life support withdrawn within 7 days. |
| Bukhari, 2020 | Aged >18 y, hemodynamically stable, admitted to ICU. | Gastrointestinal resection, contraindications for enteral feeding, history of diabetes/chronic kidney disease, receiving PN, severe intolerance, gastric residual volume >250 mL/4 h |
| Chapple, 2021 | Eligible patients were aged≥18 years; admitted to the ICU; undergoing invasive mechanical ventilation; and about to commence or had commenced EN within the preceding 12 hours and were expected to be receiving EN in the ICU until at least the calendar day after randomization. | Patients were excluded if they were expected to be receiving oral nutrition before the calendar day after randomization; if they had received any EN or PN for >12 hours during the current ICU admission; if the treating clinician considered the trial EN or rate of delivery to be clinically contraindicated; if the patient had been previously enrolled in the trial; if death was deemed imminent or inevitable during the admission; or if survival to day 90 was considered unlikely. |
| Danielis, 2019 | Adult patients (ages>18 years) admitted to the Department of Anesthesia and Intensive Care, who were undergoing mechanical ventilation within 12h from admission, and receiving parenteral or EN. | Subjects were not eligible to the study if they were malnourished (body mass index (BMI) <18.5 or≥30 kg/m^2^ ), pregnant, terminally ill, and affected by acute/chronic renal or hepatic failure. |
| Doig, 2015 | Adult patients were eligible for enrollment on day 1 or 2 of ICU stay if they were expected to remain in ICU at least 2 days after enrollment, had central venous access through which the study intervention could be delivered and were not fluid restricted to less than 1 L/day. | (1) receiving an NSAID with specific emphasis on COX-2 inhibitors；(2) currently enrolled into a clinical trial evaluating a nitric oxide (NO) inhibitor; (3) currently receiving Acetazolamide, which is a diuretic that works on the proximal tubule; (4) patient's current serum creatinine greater than the allowable age and gender adjusted maximum reported in the Maximum Creatinine Table; (5) patient have severe Acute Kidney Injury; (6) currently receiving or scheduled for dialysis / renal replacement therapy; (7) patient ever had a kidney transplant; (8) patient expected to receive palliative care only and is not expected to survive ICU or hospital discharge; (9)patient moribund and not expected to survive 24 hours; (10) patient brain dead or suspected to be brain dead. |
| Dresen, 2021 | (1) age range 18-90 years, (2) necessity of mechanical ventilation, (3) overcoming the early period of hemodynamic instability (ebb phase) according to the definitions published by ESPEN (sustainable decrease of inflammatory response assessed by lowered concentrations of procalcitonin, C-reactive protein and leukocyte numbers; decreased need for fluid resuscitation; less use of catecholamines), (4) prediction of a long-term ICU stay (valid statement by the physicians that the patient will need more than 28 days support of organ function, e.g., respiratory therapy and renal replacement therapy). | Exclusion criteria were the presence of terminal chronic renal failure, persistent acute respiratory distress syndrome (ARDS: persistent bilateral infiltrates in the chest x-ray or computed tomography scan plus ongoing compromised oxygenation defined by a need for an inspiratory oxygen fraction [FiO2] higher than 0.6), ongoing extracorporeal membrane oxygenation (ECMO), previously diagnosed myopathies, traumatic brain injuries, intracerebral hemorrhages, cerebral ischemia followed by muscular failures, and utilization of inhalation sedation. |
| Elkalawy, 2025 | Adults aged 18–50 years; Admission to the ICU within 24 hours following severe traumatic brain injury; Glasgow Coma Scale score ≤8; Injury Severity Score >15; Anticipated ICU stay of at least 4 days from enrollment; Mechanical ventilation for more than 48 hours; mNUTRIC score of 0–4; No prior history of chronic illness; Adequate baseline nutritional status; Written informed consent obtained from the patient’s surrogate. | Pregnancy; Active vascular insufficiency; Trauma involving the lower extremities; Pre-existing neuromuscular disorders; Prolonged immobility before ICU admission; Chronic renal disease; Chronic hepatic disease; Chronic cardiac disease; Diabetes mellitus; Chronic obstructive pulmonary disease; Obesity, defined as BMI ≥35 kg/m²; Active malignancy; Immunocompromised status; Recent use of high-dose corticosteroids; Use of neuromuscular blocking agents; Acute respiratory distress syndrome; Sepsis; Multiorgan failure; Complete contraindication to enteral feeding; Failure to reach 100% of the caloric target within 7 days; Persistent enteral feeding intolerance for more than 72 hours; Post-randomization exclusion following early death or failure to achieve the minimum protein intake. |
| Ferrie, 2016 | Patients in the general medical/surgical ICU of a large tertiary-referral teaching hospital requiring PN during the study period were screened for potential enrollment on their first day of PN. | Patients were excluded if they were younger than 16 years (as growth alters protein requirement), were not expected to receive at least 3 days of the intervention (eg, if expected to die, or cease PN, within 3 days), or had received significant other nutrition recently (>1 L of non-study PN solution or oral/EN providing >30% of estimated requirements in the past 3 days). |
| Fetterplace, 2018 | Patients were eligible if they were >18 years of age, mechanically ventilated for <48 hours, and were anticipated to remain ventilated for at least 72 hours. | Patients were excluded if they had a contraindication to enteral feeding, if there was futility of care or death was imminent, if they had a premorbid disability resulting in an inability to ambulate >10 m, if they were pregnant, or the treating clinician considered the intervention was not in the patient’s best interest. |
| Heyland, 2023 | Included adult patients (≥18 years) within 96 h of ICU admission who were expected to remain mechanically ventilated for at least 48 h from screening with one or more of the following nutritional risk factors: (1) low (≤25 kg/m²) or high (≥35 kg/m²) BMI; (2) moderate to severe malnutrition; (3) frailty; (4) sarcopenia; and (5) from point of screening, projected duration of mechanical ventilation of more than 4 days. | Excluded patients who had received more than 96 continuous hours of mechanical ventilation before screening, those expected to die or undergo withdrawal of life-sustaining treatments within 7 days from screening, pregnant women, patients for whom the responsible clinician felt that the patient either needed low or high protein (no clinical equipoise), and patients who required PN only in which the site did not have products to reach the high protein dose targets. |
| Kagan, 2022 | Adult patients (age 18-90 years) who had been mechanically ventilated for at least 48 h with an expected period of ventilation of a minimum of 7 days were eligible for inclusion. | Exclusion criteria were: (1) conditions that impaired the cycling movement; (2) trauma, arthritis, or surgery of the leg, pelvis, or lumbar spine; (3) open abdominal wounds or abdominal compartment syndrome; (4) an anticipated fatal outcome of ICU; (5) pre-existing diagnosis of neuromuscular weakness, acute stroke, or status epilepticus; (6) cardiorespiratory instability (need for significant vasopressor support (noradrenaline) >0.2 mcg/kg/min, dopamine >8 mcg/kg/min, inspiratory oxygen fraction (FIO2) up to 60% or PEEP (positive end expiratory pressure) up to 10 cm H2O or treatment by NO (nitric oxide), minute ventilation 150 mL/kg body weight, respiratory rate 30 breaths/min on adequate ventilatory support); (7) contra-indication for EN, including mechanical or functional bowel obstruction, high output fistula, severe necrotizing pancreatitis; (h) pregnancy. |
| Liu, 2024 | Patients to be 18 years or older, meet the diagnostic criteria for severe pneumonia according to the “Chinese Adult Community-Acquired Pneumonia Diagnosis and Treatment Guidelines (2016 Edition),” require mechanical ventilation and EN, have an expected ICU stay of more than 10 days, and have complete clinical data. | The exclusion criteria ruled out patients whose family members refused participation, those with a history of neurological or neuromuscular diseases, those with lower limb amputations, external fixators, or limb fractures, patients with severe liver or kidney impairment, pregnant patients, those who recently received nutritional support, patients with gastrointestinal diseases, obese patients (BMI>45 kg/m^2), and those with concurrent malignant tumors. The elimination criteria included subjects who requested to withdraw, patients who received EN support for less than 10 days or required PN during the study period, and patients with incomplete clinical data. |
| Miri, 2025 | Adults aged ≥18 years; Admitted to the ICU; Mechanically ventilated; Required enteral nutrition through a nasoenteric tube for at least 72 hours. | GCS score <5; Fewer than two sonographic evaluations; Unstable arm fractures; Lacerations or infections of the arm; Upper-extremity paresthesias; Upper-extremity thromboembolism; Musculoskeletal conditions; End-stage renal disease; Liver disease; Pregnancy; ICU stay shorter than 72 hours. |
| Nakamura, 2021 | Admitted to the ICU at the Hitachi General Hospital Emergency and Critical Care Center during August 2018 through September 2019 were included. | Study exclusion criteria were used to exclude patients for whom any of the following was met: younger than 20 years old; unable to use EN; lower limb injury including infection, amputation and limb ischemia; death before day 10, early expected discharge from ICU; designated as do not resuscitate or early death prognosis; CT-unable cases; extracorporeal membrane oxygenation; pacemaker implanted; inability to obtain informed consent; cases included in other clinical trials; and physician advice against patient inclusion. |
| O'Keefe, 2025 | Patients admitted to the trauma and surgical intensive care service. Receiving mechanical ventilation. Expected to require ICU care for at least 7 days. Deemed ready to start enteral nutritional support within 24–72 hours of admission. No contraindications to full enteral nutritional support. Informed consent provided by the patient or surrogate. | Severe chronic liver disease. Chronic kidney disease. Metastatic cancer. Expected to die because of the severity of illness or injury. Unable to receive full enteral nutritional support. |
| Opoda, 2024 | Mechanically ventilated patients aged 18-60 years, of both sexes, who were admitted to the surgical ICU within 1 year from July 2021 to June 2022, with an expected ICU stay≥4 days after enrollment and who were previously healthy and well-nourished (i.e., Nutric score=0). | Exclusion criteria included renal, hepatic, cardiac diseases, or COPD; previous immune abnormalities; history of nutritional problems; chronic use of drugs inducing myopathies, including treatment with corticosteroids (prednisone 40–60 mg/day for at least 2 weeks or 10 mg/ day for 4 weeks ) and muscle relaxants for more than 2 days; orthopedic problems causing immobilization of both lower limbs; amputated lower limbs or history of immobilization in the previous 2 years; history of neuromuscular diseases; a past or recent history of cancer; long-term critically ill patients who were under ventilation for more than 96 h before enrollment shifted from other hospitals; pregnant patients; severe cognitive impairment before hospitalization; open abdomen; uncontrolled status epilepticus; and intracranial hypertension. |
| Parasuraman, 2025 | Enrollment on the day of ICU admission, post-intubation status, age between 18 and 70 years inclusive, and a clinical expectation of requiring endotracheal intubation for a duration exceeding 48 h. Patients who underwent successful extubation within the initial 7-day period following intubation were subsequently excluded from the final analysis. | Patients were excluded from participation if informed written consent could not be obtained from their relative or next of kin, or if they had any of the following conditions: pregnancy, history of neurological problems, neuromuscular diseases or muscular dystrophies, vascular insufficiency or amputation of the limbs, prolonged immobility before ICU admission, unable to tolerate enteral feed, history of long-term steroids (>1 month) and trauma. |
| Reignier, 2023 | Adults (≥18 years) admitted to any of the participating ICUs were eligible if they were receiving invasive mechanical ventilation, with an expected duration of at least 48 h after inclusion and initiation either in the ICU within the past 24 h or before ICU admission having occurred within the past 24 h, concomitantly with vasoactive therapy (adrenaline, dobutamine, or noradrenaline) for shock, and if nutritional support was expected to be started within 24 h after intubation (or within 24 h after ICU admission when intubation occurred before ICU admission). | Exclusion criteria were specific nutritional needs, such as pre-existing long-term home enteral or PN for chronic bowel disease; dying patient, not-to-be-resuscitated order, or other treatment-limitation decision at ICU admission; pregnancy, recent delivery, or lactation; adult under guardianship; and correctional facility inmate. |
| Rugeles, 2013 | The study population consisted of adult patients (18 years or older) admitted in the ICU, who were expected to require EN through nasoenteric tube for at least 96 h. | Patients that had received previous nutritional support were excluded, as were patients that received concomitant PN, pregnant women, patients in transplantation program, those with contraindications for EN, chronic renal failure, uremic encephalopathy, diabetes, morbid obesity, or do-not-resuscitate orders on admission. |
| Singer, 2007 | Patients were included in the study if they were critically ill, on ventilation, required PN and had a CrC <50ml/min and furosemide-induced diuresis > 2000ml/ day. | Patients receiving dopamine or mannitol and patients in shock or hemodynamically unstable were excluded from the study. |
| Summers, 2025 | Patients were 16 years or older and prescribed EN during their index admission to the ICU or prescribed EN for the first time in the ICU during subsequent ICU admission in the index hospital admission. | Patients were excluded if the treating clinician considered the trial EN to be contraindicated or if 12 or more hours of non-trial EN had been delivered in the ICU. |
| Uyar, 2023 | Patients over the age of 18, who were expected to need mechanical ventilation, and who were hemodynamically stable were included in this study. | The exclusion criteria were patients with neuromuscular disease, spinal cord injury, septic shock, hemodynamically unstable, major abdominal and thoracic surgery, gastrointestinal injury and EN contraindications, usage of glucocorticoids, and continuous usage of neuromuscular blockers, diaphragmatic paralysis and dysfunction, mNUTRIC (Modified Nutrition Risk in Critically) score of 5 and above, high (35 kg/m2) BMI, burn, multiple trauma and pregnancy. |
| van Zanten, 2018 | Mechanically ventilated patients (aged≥18 years) admitted to the ICU, with BMI≥25; those expected to require EN starting within 48 hours after ICU admission and continuing for more than 5 days and to receive at least 800 ml of study product per day. | A Sequential Organ Failure Assessment (SOFA) score>12, any contraindication to tube nutrition or high protein tube feeds, and abnormalities in the gastrointestinal tract that could impair function. |
| Veldsman, 2025 | Adults aged ≥18 years. Admitted to the 14-bed surgical ICU of a tertiary hospital. Expected to remain in the ICU for >7 days. Required mechanical ventilation for >48 hours. Expected to receive enteral and/or parenteral nutrition for ≥5 days. Screened within the first 24–48 hours of ICU admission and enrolled within 72 hours. Informed consent obtained from the patient or initially by telephone from the next of kin, followed by written consent where applicable. | Preexisting muscle weakness. Any condition rendering muscle biopsy unsafe. Any condition precluding safe in-bed cycling. Any condition making intravenous amino acid supplementation inappropriate. Lower-limb fractures or amputations. Body mass index >35 kg/m². New or worsening renal failure not requiring renal replacement therapy.Acute kidney injury of KDIGO stage ≥2. |
| Verceles, 2023 | Older >50 year-old, mechanically ventilated participants (≥24 hours) were screened daily using the following eligibility criteria: pre-admission Barthel Index of ≥70, ability to follow commands and able to perform physical therapy testing prior to ICU admission. | NA |
| Wang, 2024 | Eligibility criteria included critically ill patients aged above 18 years admitted to ICU and EICU. Patients with mNUTRIC score>5 and an anticipated ICU stay ICU/ EICU of more than 7 days were included. | Patients with the following characteristics were excluded from the study: (1) burns; (2) inability to initiate EN within 48 h of ICU admission; (3) initiation of renal replacement therapy within 24–48 h of ICU/ EICU admission; (4) pregnancy or lactation; (5) advanced malignant tumors; (6) severe complications of diabetes such as ketoacidosis, hyperosmolar coma, or acidosis; (7) a history of digestive tract surgery, primary gastrointestinal injury or neuromuscular diseases (affecting protein absorption or metabolism); (8) BMI≥30 kg/m2 ; (9) hyperthyroidism; (10) unwillingness to participate in the study. |
| Zhang, 2022 | Patients≥18 years old who had respiratory failure due to various causes and required tracheal intubation and mechanical ventilation, which was maintained for at least 7 days, and whose conditions were reversible. | Patients with (1) neuromuscular disease; (2) spinal cord injury; (3) severe thoracic deformity and respiratory restriction; (4) gastrointestinal injury and EN contraindications; (5) moderate to severe hepatic insufficiency；(6) usage of glucocorticoids and continuous usage of neuromuscular blockers; and (7) pregnancy, as well as (8) patients for whom clinicians considered the intervention not to be in the patient’s best interest. |

Note: ICU, intensive care unit; EN, enteral nutrition; PN, parenteral nutrition; ALI, Acute Lung Injury; TBI, traumatic brain injury; GCS, Glasgow Coma Scale; NSAID, non-steroidal anti-inflammatory drugs; COPD, chronic obstructive pulmonary disease; mNUTRIC, Modified Nutrition Risk in Critically; BMI, body mass index; EICU, emergency ICU.

|  | | | | | | | | | | | | | | | | |
| --- | --- | --- | --- | --- | --- | --- | --- | --- | --- | --- | --- | --- | --- | --- | --- | --- |
| Table S4: Demographic details of the included RCTs. | | | | | | | | | | | | | | | | |
| Author, year | Protein (high/ low) | Age | Male  (n, %) | BMI | Actual body weight (kg) | Ideal body weight  (kg) | APACHE II | SOFA | GCS | Clinical Frailty Score | NUTRIC Score | MV (n,%) | Vasopressor therapy (n,%) | Renal replacement therapy (n,%) | Calculated caloric requirement, kcal/d | Calculated protein requirement, g/kg/d |
| Ahmadpour, 2020 | High | 47±18 | 7 (46%) | 28±14 | NA | NA | 24±4.5 | NA | 6.2±1.7 | NA | NA | NA | NA | NA | NA | NA |
|  | Low | 53±21 | 7 (46%) | 28±5.5 | NA | NA | 23±5.7 | NA | 7±2.9 | NA | NA | NA | NA | NA | NA | NA |
| Azevedo, 2019 | High | 65±18.8 | 34(60%) | NA | NA | NA | 81.1±32.4 | 9.8±14.6 | NA | NA | NA | NA | NA | NA | 1554(1383-1862) | 2.1(2.1-2.1) |
|  | Low | 67.4±18.9 | 32(51%) | NA | NA | NA | 77.2±30.7 | 6.8±4 | NA | NA | NA | NA | NA | NA | 1450(1300-1625) | 1.5(1.5-1.5) |
| de Azevedo, 2021 | High | 67.6±17.8 | 34(61%) | NA | NA | NA | NA | 5(3–9) | NA | NA | NA | NA | NA | NA | NA | NA |
|  | Low | 65.3±19.7 | 48(49%) | NA | NA | NA | NA | 6(3.7–8) | NA | NA | NA | NA | NA | NA | NA | NA |
| Badjatia, 2021 | High | 60±8 | 5(42%) | 27 ± 3 | NA | NA | 20±5 | NA | NA | NA | NA | NA | NA | NA | NA | NA |
|  | Low | 58±14 | 5(38%) | 27 ± 6 | NA | NA | 18±10 | NA | NA | NA | NA | NA | NA | NA | NA | NA |
| Badpeyma, 2025 | High | 54.3±19.3 | 15(54%) | 21.6±4.5 | 60±16 | NA | 20.4±5.2 | 6.4±1.7 | NA | 3.7±2.2 | NA | 28(100%) | NA | NA | NA | 2.2 |
|  | Low | 58.2±18.6 | 18(64%) | 23±3.3 | 64.9±10 | NA | 21.3±5.3 | 7±3.1 | NA | 4.8±3.2 | NA | 28(100%) | NA | NA | NA | 1.0 |
| Bels, 2024 | High | 62±14 | 291(62%) | 28±6 | NA | NA | 21±7 | 9±3 | 15(8–15) | 3±2 | NA | NA | NA | NA | NA | NA |
|  | Low | 63±14 | 309(67%) | 27±5 | NA | NA | 22±7 | 10±3 | 15(6–15) | 3±2 | NA | NA | NA | NA | NA | NA |
| Bibi,2025 | High | 64.4±11.6 | 30(70%) | 27±6.4 | NA | NA | 17.8±4.4 | 13.7±1.6 | 13.7±1.6 | NA | 5.5±1.4 | NA | NA | NA | NA | NA |
|  | Low | 61±3.5 | 28(62%) | 26.7±4.8 | NA | NA | 15.5±4 | 6.7±1.8 | 13.1±2.3 | NA | 5.8±1.5 | NA | NA | NA | NA | NA |
| Bukhari, 2020 | High | 42.7±18 | NA | NA | NA | 56.8±7.1 | NA | NA | NA | NA | NA | NA | NA | NA | NA | NA |
|  | Low | 41.8±19.4 | NA | NA | NA | 55.9±4.4 | NA | NA | NA | NA | NA | NA | NA | NA | NA | NA |
| Chapple, 2021 | High | 60(50-72) | 39(67%) | 29(26-33) | 85(75-100) | 65(57-71) | 22(16-26) | NA | NA | NA | NA | NA | 37(64%) | 4(7%) | 1882(1503-2050) | 1.46(1.31-1.58) |
|  | Low | 61(46-68) | 44(76%) | 30(25-34) | 85(75-101) | 67(57-72) | 22(16-27) | NA | NA | NA | NA | NA | 37(64%) | 2(3%) | 1900(1628–2075) | 1.47(1.38-1.57) |
| Danielis, 2019 | High | 66(57-72) | 11(58%) | 25(23-28) | NA | NA | 17(14-22) | 32(28-49) | NA | NA | NA | 12(63%) | NA | NA | NA | NA |
|  | Low | 63(46-70) | 11(52%) | 24(21-26) | NA | NA | 17(13-22) | 40(31-48) | NA | NA | NA | 11(52%) | NA | NA | NA | NA |
| Doig, 2015 | High | 63.3±15.4 | 158(66%) | 28.9±7 | NA | NA | 21.7±7.6 | NA | NA | NA | NA | 197 (82%) | NA | NA | NA | NA |
|  | Low | 62.7±16.6 | 147(63%) | 29.5±6.9 | NA | NA | 20.2±6.8 | NA | NA | NA | NA | 194 (83%) | NA | NA | NA | NA |
| Dresen, 2021 | High | 66±16 | 15(71%) | NA | NA | 75±8 | NA | 6±3 | NA | NA | NA | NA | NA | NA | NA | NA |
|  | Low | 64±15 | 15(71%) | NA | NA | 78±10 | NA | 8±3 | NA | NA | NA | NA | NA | NA | NA | NA |
| Elkalawy, 2025 | High | 40±6 | 23(43.4%) | 30.05±1.77 | 78±5.25 | NA | NA | NA | NA | NA | 3(2–3) | 53(100%) | NA | NA | NA | 2.2 |
|  | Low | 39±7 | 28 (57.1%) | 30.45±1.79 | 79±5.32 | NA | NA | NA | NA | NA | 3 (2–3) | 49(100%) | NA | NA | NA | 1.5 |
| Ferrie, 2016 | High | 67(56-74) | 38(63%) | NA | 73.2±16.1 | NA | 25.5±9.4 | 9.4±4.1 | NA | NA | 5.8±2.1 | 56(95%) | NA | 9(15%) | NA | NA |
|  | Low | 65(49-70) | 36(60%) | NA | 77.7±21.7 | NA | 23.7±8.1 | 9.2±4 | NA | NA | 5.4±2.2 | 59(98%) | NA | 11(18%) | NA | NA |
| Fetterplace, 2018 | High | 55±13 | 23(77%) | 30±7.1 | NA | NA | 22±6.2 | NA | NA | NA | NA | NA | NA | NA | 1969±277 | 1.3±0.1 |
|  | Low | 57±16 | 21(70%) | 29±5.3 | NA | NA | 20±5.9 | NA | NA | NA | NA | NA | NA | NA | 1918±255 | 1.3±0.1 |
| Heyland, 2023 | High | 57(18-95) | 395(61%) | 28(13-85) | NA | NA | 21(16-27) | 9(6-11) | NA | 3(2-5) | 5(3-6) | NA | 254(39%) | 77(12%) | NA | NA |
|  | Low | 57(18-93) | 388(59%) | 29(13-77) | NA | NA | 21(15-26) | 9(6-11) | NA | 3(2-4) | 5(3-6) | NA | 266(41%) | 58(9%) | NA | NA |
| Kagan, 2022 | High | 61±16 | 14(73.7%) | NA | NA | NA | 22±8 | 7±4 | NA | NA | NA | NA | NA | NA | NA | NA |
|  | Low | 63±18 | 12(57.1%) | NA | NA | NA | 20±6 | 5±2 | NA | NA | NA | NA | NA | NA | NA | NA |
| Liu, 2024 | High | 61.2±12 | 39(65%) | 22.9±2.9 | 64.2±10.6 | 60.2±3.7 | 19.2±5.5 | 6.4±2.4 | NA | NA | NA | NA | NA | NA | NA | NA |
|  | Low | 61.7±12.2 | 41(68.3%) | 22.8±3 | 63.8±10.6 | 60.0±3.9 | 20.7±5.9 | 6.9±3.2 | NA | NA | NA | NA | NA | NA | NA | NA |
| Miri, 2025 | High | 51.5±14.2 | 25(50.0%) | 21.6±2.8 | NA | NA | 17.7±6 | 8.4±2.9 | NA | NA | NA | 50(100%) | NA | NA | NA | 1.5 |
|  | Low | 52.2±10.8 | 29(58.0%) | 20.9±2.7 | NA | NA | 17.4±6 | 8.1±3.2 | NA | NA | NA | 50(100%) | NA | NA | NA | 1.0 |
| Nakamura, 2021 | High | 68.3±14.3 | 35(58%) | 21.3±3.9 | 53.1±9.6 | NA | 18.6±8.1 | 6.8±3.1 | NA | NA | NA | 51(85%) | 31(53%) | 9(17%) | NA | NA |
|  | Low | 67.9±14.9 | 38(67%) | 21.5±4.5 | 53.6±11.2 | NA | 18.2±6 | 7.1±3.5 | NA | NA | NA | 52(93%) | 34(61%) | 8(16%) | NA | NA |
| O'Keefe, 2025 | High | 45.1±16.5 | 202(79%) | 28.1±7.2 | NA | NA | 26.7±7.1 | NA | NA | NA | NA | 244(100%) | NA | NA | 25 | 2 |
|  | Low | 47.4±17.4 | 184(75%) | 28.7±7.9 | NA | NA | 27.7±7.1 | NA | NA | NA | NA | 256(100%) | NA | NA | 25 | NA |
| Opoda, 2024 | High | 32.5±8.3 | 25 (69%) | NA | NA | 64.6±10.8 | NA | 3(2.5-4) | NA | NA | NA | NA | NA | NA | NA | NA |
|  | Low | 30.8±9.7 | 20 (59%) | NA | NA | 64.2±8.2 | NA | 3(3-4) | NA | NA | NA | NA | NA | NA | NA | NA |
| Parasuraman, 2025 | High | 35.8±13.7 | 9 (60%) | NA | 63.6±8.9 | NA | 15.7±7.3 | 7.0±2.8 | NA | NA | NA | NA | NA | NA | NA | NA |
|  | Low | 36.6±13.9 | 8 (53%) | NA | 63.5±9 | NA | 17.8±5.1 | 8.9± 2.7 | NA | NA | NA | NA | NA | NA | NA | NA |
| Reignier, 2023 | High | 66±13 | 1026(68%) | 27(23-32) | 77(65-90) | NA | NA | 10(8-13) | NA | NA | NA | NA | NA | 183(12%) | NA | NA |
|  | Low | 66±13 | 1010(66%) | 27(23-31) | 77(65-90) | NA | NA | 10(8-13) | NA | NA | NA | NA | NA | 161(11%) | NA | NA |
| Rugeles, 2013 | High | 53.3±19.5 | 22(55%) | 23.7±3.3 | 63.0±10.7 | NA | 13.9±4.8 | 7.5±2.9 | NA | NA | NA | NA | NA | NA | NA | NA |
|  | Low | 55.7±19.5 | 24(60%) | 24.3±4.4 | 65.8±11 | NA | 15.1±6.2 | 6.7±2.5 | NA | NA | NA | NA | NA | NA | NA | NA |
| Singer, 2007 | High | 74.1±5.7 | NA | NA | NA | NA | NA | NA | NA | NA | NA | NA | NA | NA | NA | NA |
|  | Low | 63±15.6 | NA | NA | NA | NA | NA | NA | NA | NA | NA | NA | NA | NA | NA | NA |
| Summers, 2025 | High | 61(48-70) | 1070(64%) | 28(24-33) | 80(68-94) | 66(57-73) | 19(15-24) | NA | NA | 3(3-4) | NA | 1385(82%) | 926(55%) | 122(7%) | NA | NA |
|  | Low | 61(48-71) | 1087(63%) | 28(24-32) | 80(67-97) | 66(57-73) | 19(14-25) | NA | NA | 3(3-4) | NA | 1355(79%) | 921 (53.7) | 119(7%) | NA | NA |
| Uyar, 2023 | High | 61.7±22 | 19(76%) | 22.2±3.9 | NA | NA | 30.5±11.3 | 8.1±3.2 | NA | NA | NA | NA | NA | NA | NA | NA |
|  | Low | 59.8±23.8 | 12(50%) | 22.8±4.4 | NA | NA | 33.2±8.6 | 7.5±2.3 | NA | NA | NA | NA | NA | NA | NA | NA |
| van Zanten, 2018 | High | 60.8±15.2 | 13(59%) | 30.7±8.4 | 91.2±20.7 | NA | 24(18-27) | 9(7-11) | NA | NA | NA | NA | NA | NA | NA | NA |
|  | Low | 63.9±13.3 | 9(41%) | 30.3±4.1 | 84.9±18.3 | NA | 25(21-28) | 10(9-11) | NA | NA | NA | NA | NA | NA | NA | NA |
| Veldsman, 2025 | High | 37.4±11.9 | 23(92%) | 24.8±3.9 | 71.6±13.8 | NA | 13(9-17.5) | 4.7±2.9 | NA | NA | 2(1-3) | 25(100%) | NA | NA | NA | 1.3+0.25-0.4 |
|  | Low | 36.4±11.8 | 22(88%) | 24.7±4.2 | 73.6±13.3 | NA | 12(9-17.5) | 4.4±3.5 | NA | NA | 1(1-2.5) | 25(100%) | NA | NA | NA | 1.3 |
| Verceles, 2023 | High | 62±9.3 | 8(50%) | 29±5.5 | NA | NA | 17.8±5.5 | NA | NA | NA | NA | NA | NA | NA | NA | NA |
|  | Low | 62±9.3 | 10(43%) | 30±6.6 | NA | NA | 15.4±7.4 | NA | NA | NA | NA | NA | NA | NA | NA | NA |
| Wang, 2024 | High | 76(64-83) | 55(64%) | 22(19-24) | 67.2±8.2 | NA | 23(16-26) | NA | NA | NA | 7(6-7) | NA | NA | NA | NA | NA |
|  | Low | 75(65-82) | 56(64%) | 22(20-25) | 66±11.8 | NA | 22(18-26) | NA | NA | NA | 7(6-8) | NA | NA | NA | NA | NA |
| Zhang, 2022 | High | 64.5±16.2 | 12(60%) | 22.2±3.9 | NA | NA | 21.8±7.2 | 8.2±3.1 | NA | NA | NA | NA | NA | NA | NA | NA |
|  | Low | 69.2±18.2 | 18(86%) | 22.8±4.4 | NA | NA | 20.5±7 | 8.1±2.4 | NA | NA | NA | NA | NA | NA | NA | NA |

Note: Data were n (%), mean ± SD, or median (IQR); * g/d; NA, not available.

| Table S5: Admission reason of the included RCTs. | | | | | | | | | | | |
| --- | --- | --- | --- | --- | --- | --- | --- | --- | --- | --- | --- |
|  |  | Admission reason | | | | | | | | Admission category | |
| Author, year | Protein | Sepsis | Neurological | Cardiovascular | Respiratory | Gastrointestinal | Trauma | Renal | Others | Medical | Surgical |
| Ahmadpour, 2020 | High | NA | NA | NA | NA | NA | NA | NA | NA | NA | NA |
|  | Low | NA | NA | NA | NA | NA | NA | NA | NA | NA | NA |
| Azevedo, 2019 | High | 12 (21.0%) | 11 (19.2%) | 17 (29.8%) | 9 (15.7%) | 4 (7.0%) | NA | NA | 4 (7.0%) | 46 (80%) | 11 (20%) |
|  | Low | 15 (23.8%) | 9 (14.2%) | 23 (36.5%) | 11 (17.4%) | 4 (6.3%) | NA | NA | 1 (1.5%) | 46 (73%) | 17 (27%) |
| Azevedo, 2021 | High | NA | 17 (20%) | 6 (7%) | 28 (32%) | 9 (10%) | 6 (7%) | 3 (4%) | 15 (17%) | 64 (73%) | 23 (27%) |
|  | Low | NA | 17 (20%) | 8 (10%) | 26 (30%) | 8 (9%) | 4 (5%) | 2 (3%) | 20 (23%) | 70 (74%) | 24 (26%) |
| Badjatia, 2021 | High | NA | NA | NA | NA | NA | NA | NA | NA | NA | NA |
|  | Low | NA | NA | NA | NA | NA | NA | NA | NA | NA | NA |
| Badpeyma, 2025 | High | 3 (10.7%) | 3 (10.7%) | 1 (3.6%) | 7 (25.0%) | NA | NA | NA | 14 (50.0%) | NA | NA |
|  | Low | 5 (17.9%) | 6 (21.4%) | 3 (10.7%) | 6 (21.4%) | NA | NA | NA | 8 (28.6%) | NA | NA |
| Bels, 2024 | High | 26 (6%) | 82 (17%) | 129 (27%) | 149 (32%) | 22 (5%) | 48 (10%) | NA | 14(3%) | 308 (66%) | 162 (35%) |
|  | Low | 21 (5%) | 67 (14%) | 128 (28%) | 173 (37%) | 19 (4%) | 42 (9%) | NA | 15(3%) | 341 (73%) | 124 (27%) |
| Bibi,2025 | High | NA | 4 (9.3%) | 2 (4.7%) | 27 (62.8%) | 2 (4.7%) | NA | 2 (4.7%) | 6 (14.0%) | NA | NA |
|  | Low | NA | 3 (6.7%) | 4 (8.9%) | 30 (66.7%) | 0 (0%) | NA | 4 (8.9%) | 4 (8.9%) | NA | NA |
| Bukhari, 2020 | High | NA | NA | NA | NA | NA | NA | NA | NA | NA | NA |
|  | Low | NA | NA | NA | NA | NA | NA | NA | NA | NA | NA |
| Chapple, 2021 | High | NA | NA | NA | NA | NA | NA | NA | NA | 47 (81%) | 11 (20%) |
|  | Low | NA | NA | NA | NA | NA | NA | NA | NA | 41 (71%) | 17 (30%) |
| Danielis, 2019 | High | NA | NA | NA | NA | 4 (21%) | 5 (26%) | NA | 7 (37%) | NA | 3 (16%) |
|  | Low | NA | NA | NA | NA | 7 (33%) | 3 (14%) | NA | 9 (43%) | NA | 2 (10%) |
| Doig, 2015 | High | 35 (14.6%) | 29 (12.1%) | 52 (21.8%) | 60 (25.1%) | 41 (17.2%) | 15 (6.3%) | 0 (0%) | 7 (2.9%) | 159 (85.6%) | 80 (14.4%) |
|  | Low | 25 (10.6%) | 18 (7.7%) | 48 (20.4%) | 63 (26.8%) | 49 (20.9%) | 17 (7.2%) | 1 (0.4%) | 14 (5.9%) | 156 (66.4%) | 79 (33.6)5 |
| Dresen, 2021 | High | 19 (90%) | NA | 5 (24%) | NA | 5 (24%) | 3 (14%) | 3 (14%) | NA | NA | 2 (10%) |
|  | Low | 17 (81%) | NA | 2 (10%) | NA | 5 (24%) | 1 (4%) | 8 (38%) | NA | NA | 5 (24%) |
| Elkalawy, 2025 | High | 0 | NA | 0 | 0 | 0 | 53 (100%) | 0 | 0 | NA | NA |
|  | Low | 0 | NA | 0 | 0 | 0 | 49 (100%) | 0 | 0 | NA | NA |
| Ferrie, 2016 | High | NA | NA | NA | NA | NA | NA | NA | NA | NA | NA |
|  | Low | NA | NA | NA | NA | NA | NA | NA | NA | NA | NA |
| Fetterplace, 2018 | High | 3 (10%) | 13 (43%) | 3 (10%) | 1 (3.3%) | NA | 7 (23%) | 1 (3.3%) | 3 (10%) | 21 (70%) | 9 (30.3%) |
|  | Low | 1 (3.3%) | 7 (23%) | 3 (10%) | 5 (17%) | NA | 11 (37%) | 0 (0%) | 3 (10%) | 16 (53%) | 14 (46.3%) |
| Heyland, 2023 | High | 81 (13%) | 104 (16%) | 56 (9%) | 275 (43%) | 26 (4%) | 59 (9%) | NA | 23 (4%) | 548 (85%) | 97 (15%) |
|  | Low | 90 (14%) | 93 (14%) | 52 (8%) | 274 (42%) | 37 (6%) | 67 (10%) | NA | 17 (3%) | 540 (82%) | 116 (18%) |
| Kagan, 2022 | High | 4 (21%) | NA | 6 (32%) | 5 (26%) | NA | 4 (21%) | NA | NA | NA | NA |
|  | Low | 3 (14%) | NA | 2 (10%) | 10 (48%) | NA | 4 (19%) | NA | NA | NA | NA |
| Liu, 2024 | High | NA | NA |  | NA | NA | NA | NA | NA | NA | NA |
|  | Low | NA | NA | NA | NA | NA | NA | NA | NA | NA | NA |
| Miri, 2025 | High | 2 (4.0%) | 24 (48.0%) | 4 (8.0%) | NA | NA | 6 (12.0%) | NA | 7 (14.0%) | 6 (12.0%) | 1 (2.0%) |
|  | Low | 2 (4.0%) | 16 (32.0%) | 0 (0.0%) | NA | NA | 9 (18.0%) | NA | 16 (32.0%) | 3 (6.0%) | 4 (8.0%) |
| Nakamura, 2021 | High | 27 (45.0%) | 29 (48.3%) | 24 (40.0%) | 21 (35.0%) | NA | 3 (5.0%) | 8 (13%) | NA | NA | 21 (35.6%) |
|  | Low | 30 (52.6%) | 31 (54.4%) | 21 (36.8%) | 13 (22.8%) | NA | 7 (12.3%) | 8 (14%) | NA | NA | 25 (43.9%) |
| O'Keefe, 2025 | High | NA | NA | NA | NA | NA | 223 (87%) | NA | 33 (13%) | NA | 244 (100%) |
|  | Low | NA | NA | NA | NA | NA | 207 (85%) | NA | 37 (15%) | NA | 256 (100%) |
| Opoda, 2024 | High | NA | 6 (16.7%) | NA | NA | 6 (16.7%) | 24 (66.7% | NA | NA | NA | NA |
|  | Low | NA | 5 (14.7%) | NA | NA | 8 (23.5%) | 21 (61.7%) | NA | NA | NA | NA |
| Parasuraman, 2025 | High | 11 (73.3%) | 3 (20%) | NA | 5 (33.3%) | NA | NA | NA | 7(47%) | NA | NA |
|  | Low | 10 (66.7%) | 0 (0%) | NA | 8 (53.33%) | NA | NA | NA | 7(47%) | NA | NA |
| Reignier, 2023 | High | 874 (57.7%) | 112 (7.4%) | 462 (30.5%) | 654 (43.2%) | NA | 28 (1.8%) | NA | 258 (18.3%) | 1258 (83.1%) | NA |
|  | Low | 889 (58.7%) | 109 (7.2%) | 417 (27.5%) | 686 (45.3%) | NA | 25 (1.7%) | NA | 278 (17.7%) | 1253 (82.8%) |  |
| Rugeles, 2013 | High | NA | 13 (33%) | 2 (5%) | 16 (40%) | 0 (0%) | NA | NA | 9 (23%) | NA | NA |
|  | Low | NA | 12 (30%) | 4 (10%) | 14 (35%) | 3 (8%) | NA | NA | 7 (18%) | NA | NA |
| Singer, 2007 | High | NA | NA | NA | NA | NA | NA | NA | NA | NA | NA |
|  | Low | NA | NA | NA | NA | NA | NA | NA | NA | NA | NA |
| Summers, 2025 | High | 148 (8.8%) | 374 (22.2%) | 300 (17.8%) | 325 (19.3%) | 168 (10.0%) | 204 (12.1%) | 162 (9.6%) | NA | 1068 (64%) | 613(36%) |
|  | Low | 134 (7.8%) | 350 (20.4%) | 310 (18.1%) | 360 (21.0%) | 190 (11.1%) | 209 (12.2%) | 163 (9.5%) | NA | 1100 (64%) | 586(36%) |
| Uyar, 2023 | High | 0 (0%) | 6 (24.0%) | 1 (4.0%) | 14 (56.0%) | NA | 1 (4.0%) | 3 (12.0%) | 0 (0%) | NA | NA |
|  | Low | 2 (8.3%) | 3 (12.5%) | 1 (4.2%) | 13 (54.2%) | NA | 1 (4.2%) | 2 (8.3%) | 2 (8.3%) | NA | NA |
| van Zanten, 2018 | High | NA | NA | NA | NA | NA | 9 (40.9%) | NA | NA | 9 (40.9%) | 10 (45.5%) |
|  | Low | NA | NA | NA | NA | NA | 10 (45.5%) | NA | NA | 8 (36.4%) | 11 (50.0%) |
| Veldsman, 2025 | High | NA | NA | NA | 1 (4%) | 5 (20%) | 19 (76%) | NA | NA | 2 (8%) | 23 (92%) |
|  | Low | NA | NA | NA | 2 (8%) | 4 (16%) | 19 (76%) | NA | NA | 3 (12%) | 22 (88%) |
| Verceles, 2023 | High | NA | 0 (0%) | 0 (0%) | 13 (81%) | NA | NA | NA | 3 (19%) | NA | NA |
|  | Low | NA | 1 (4%) | 0 (0%) | 19 (83%) | NA | NA | NA | 3 (13%) | NA | NA |
| Wang, 2024 | High | 20 (23.3%) | 16 (18.6%) | NA | 37 (43.0%) | 22 (25.6%) | NA | 10 (11.6%) | 1 (1.2%) | NA | NA |
|  | Low | 15 (17.2%) | 11 (12.6%) | NA | 41 (47.1%) | 22 (25.3%) | NA | 12 (13.8%) | 1 (1.2%) | NA | NA |
| Zhang, 2022 | High | 13 (65%) | NA | NA | NA | NA | NA | NA | NA | 8 (40%) | 12 (60%) |
|  | Low | 16 (76.2%) | NA | NA | NA | NA | NA | NA | NA | 9 (42.9%) | 12 (57.1%) |

Note: Data were n (%); NA, not available.

Table S6. Reported and selected outcome time points

| **Outcomes** | **Selected time point** | **Outcomes** | **Reported time point** | **Selected time point** | **Outcomes** | **Reported time point** | **Selected time point** |
| --- | --- | --- | --- | --- | --- | --- | --- |
| **all-cause mortality** | **Longest follow-up** | **Atrophy rate of thigh** **muscle thickness** |  |  | **Nitrogen balance** |  |  |
| Ahmadpour, 2020 | 28-day Mortality | Dresen, 2021 | 1,14,28-day | 28-day | Ahmadpour, 2020 | 1,5-day | 5-day |
| Azevedo,2019 | Hospital Mortality | Liu, 2024 | 1,5,10-day | 10-day | Badjatia, 2021 | 2-4,7-9,12-14 day | 12-14 day |
| Azevedo, 2021 | 180-day Mortality | Nakamura, 2021 | 1,10-day | 10-day | Bibi,2025 | ICU discharge | ICU discharge |
| Badjatia, 2021 | 90-day Mortality | Opoda, 2024 | 1,5,10,20,30-day | 30-day | Danielis, 2019 | 1,3-day, study exit | Study exit |
| Badpeyma, 2025 | 60-day Mortality | Parasuraman, 2025 | 1,3,5,7-day | 7-day | Ferrie, 2016 | 3,7-day | 7-day |
| Bels, 2024 | 90-day Mortality | Uyar, 2023 | 1,3,5,7,15-day | 15-day | Miri, 2025 | 3,5-day | 5-day |
| Bukhari, 2020 | Hospital Mortality | Wang, 2024 | 1,5-day, ICU discharge | ICU discharge | Singer, 2007 | 1,2,3,4-day | 4-day |
| Chapple, 2021 | 90-day Mortality | **Atrophy rate of thigh muscle CSA** |  |  | Verceles, 2023 | 1,5,9,14-day | 14-day |
| Danielis, 2019 | Mortality after ICU discharge | Badjatia, 2021 | 0-2,14-day | 14-day | Zhang, 2022 | 1,2,3,4,5-week | 5-week |
| Doig, 2015 | 90-day Mortality | Elkalawy, 2025 | 0,5,10,15,20-day | 20-day | **Albumin** |  |  |
| Dresen, 2021 | ICU Mortality | Opoda, 2024 | 1,5,10,20,30-day | 30-day | Badpeyma, 2025 | 1,12-day | 12-day |
| Ferrie, 2016 | 180-day Mortality | Parasuraman, 2025 | 1,3,5,7-day | 7-day | Elkalawy, 2025 | 0,5,10,15,20-day | 20-day |
| Fetterplace, 2018 | 60-day Mortality | Veldsman, 2026 | 2,8-day | 8-day | Fetterplace, 2018 | ICU discharge or day 15 | ICU discharge or day 15 |
| Heyland, 2023 | 60-day Mortality | Verceles, 2023 | 7,14-day | 14-day | Liu, 2024 | 1,5,10-day | 10-day |
| Kagan, 2022 | Hospital Mortality | Wang, 2024 | 1,5-day, ICU discharge | ICU discharge | Nakamura, 2021 | 10-day | 10-day |
| Miri, 2025 | During the study period | **Absolute change in MUAC** |  |  | Summers, 2025 | 10-day | 10-day |
| Nakamura, 2021 | 28-day Mortality | Badpeyma, 2025 | 1,12-day | 12-day | **Urea** |  |  |
| O'Keefe, 2025 | Hospital Mortality | Fetterplace, 2018 | ICU discharge or day 15 | ICU discharge or day 15 | Badpeyma, 2025 | 1,12-day | 12-day |
| Opoda, 2024 | 30-day Mortality | Miri, 2025 | 3,6,9,12,15,18,21-day | 21-day | Chapple, 2021 | Every day | Mean daily level |
| Reignier, 2023 | 90-day Mortality | **Atrophy rate of** **diaphragm** |  |  | Heyland, 2023 | 12-day | Mean daily level |
| Singer, 2007 | Mortality after ICU discharge | Liu, 2024 | 1,5,10-day | 10-day | Summers, 2025 | 10-day | 10-day |
| Summers, 2025 | 90-day Mortality | Uyar, 2023 | 1,3,5,7,15-day | 15-day | Veldsman, 2026 | 0,10-day | 10-day |
| Uyar, 2023 | 28-day Mortality | Zhang, 2022 | 2,3,4,5-week | 5-week | **Creatinine** |  |  |
| van Zanten, 2018 | 30-day Mortality | **Handgrip strength** |  |  | Badpeyma, 2025 | 1,12-day | 12-day |
| Wang, 2024 | 28-day Mortality | Azevedo,2019 | Hospital discharge | Hospital discharge | Chapple, 2021 | Every day | Mean daily level |
| Zhang, 2022 | ICU Mortality | Ferrie, 2016 | 7-day, Hospital discharge | Hospital discharge | Danielis, 2019 | 1,3-day, study exit | Study exit |
|  |  | Fetterplace, 2018 | ICU discharge or day 15 | ICU discharge or day 15 | Heyland, 2023 | 12-day | Mean daily level |
|  |  | **HRQoL** |  |  | Nakamura, 2021 | 10-day | 10-day |
|  |  | Bels, 2024 | 30,90,180-day | 30-day | O'Keefe, 2025 | 1,2,3-week | 3-week |
|  |  | Chapple, 2021 | 90-day | 90-day | Singer, 2007 | 1,2,3,4-day | 4-day |
|  |  | Nakamura, 2021 | Hospital discharge | Hospital discharge | Veldsman, 2026 | 0,4,7,10-day | 10-day |
|  |  |  |  |  | **BUN** |  |  |
|  |  |  |  |  | Elkalawy, 2025 | 0,5,10,15,20-day | 20-day |
|  |  |  |  |  | Nakamura, 2021 | 10-day | 10-day |
|  |  |  |  |  | O'Keefe, 2025 | 1,2,3-week | 3-week |
|  |  |  |  |  | Singer, 2007 | 1,2,3,4-day | 4-day |
|  |  |  |  |  | van Zanten, 2018 | 5-day | 5-day |

Table S7. Details of physical rehabilitation

| Author, year | | Intervention type | Device | Details | Duration |
| --- | --- | --- | --- | --- | --- |
| Badjatia, 2021 | Common intervention for both groups | Basic mobilization |  | Started from the first day after aneurysm securing procedure, guided by nurses or therapists, including transfer from bed to chair, ambulation with assistance as tolerated; daily recording of activities; both groups had equal access to therapists. | Up to 14 days |
|  | High protein group | NMES (neuromuscular electrical stimulation) | L300 Plus® system | With bilateral thigh cuffs to stimulate quadriceps; 2 sessions daily, 30 minutes each (09:00-11:00 and 13:00-17:00); amplitude titrated to achieve visible muscle contraction without pain, performed in supine position (legs partially flexed) or sitting in bedside chair. |  |
|  | Low protein group | no NMES |  | No neuromuscular electrical stimulation, only basic mobilization. |  |
| Azevedo, 2021 | High protein group | Early exercise | Moto Med Letto II cycle ergometer(ReckTechnik,Germany) | Cycle ergometry exercise, 2 sessions daily, 15 minutes each; resistance gradually increased during the first week. | Started immediately after randomization and continued until discharge, death, or day 21 of the study (whichever came first). |
|  | Low protein group | Routine physiotherapy |  | Included early bed activity and passive and active movements at least twice a day, following the ICU's routine physiotherapy protocol. |  |
| Kagan, 2022 | High/ Low protein group | Cycle ergometry exercise (CE) | MOTOmed viva2 device (Medimotion, UK) | for conscious patients able to cycle actively, sessions divided into two 10-minute bouts or more intervals; for sedated patients, 20 consecutive minutes of passive cycling; training intensity evaluated and vital signs recorded at the start, 10 minutes after, and end of each session, with patients in a comfortable position between supine and semi-recumbent. | Started within 24 hours after randomization, continued until ICU discharge, death, or 28 days (whichever came first). |
| Nakamura, 2021 | High/ Low protein group | Former period: standard rehabilitation training, without EMS |  | August 2018 to March 2019, without EMS: Physical therapists started from day 2, 20 minutes daily, including range of motion exercises, kicking a stability ball, standing exercise, and ambulation exercise, conducted on the bed or at the bedside based on the patient's condition; nurses provided additional physical therapy (following medical orders). | Continued until day 10 |
|  |  | Latter period: Standard rehabilitation training + electrical muscle stimulation (EMS) | belt-type EMS (G-TES®) | April 2019 to September 2019, with EMS: In addition to the above standard rehabilitation training, the device covering the abdomen and all lower extremities was added from day 2; 1 session daily, 20 minutes each, with a frequency of 20Hz, pulse width of 250ms (5 seconds of stimulation followed by 2 seconds of rest), intensity adjusted by physical therapists; nurses provided additional physical therapy. | Continued until day 10. |
| Opoda, 2024 | High protein group | Protocolized physical exercise |  | Level I (unconscious mechanically ventilated patients): 3 times daily, 7 days a week, passive range of motion training for upper and lower limb joints (5 repetitions per joint), 20 minutes per session (starting with 10 minutes and gradually increasing), combined with usual care; Level II (conscious and responsive patients): active range of motion training, resisted range of motion training, bed exercises, sitting on the edge of the bed, standing, walking, etc., combined with usual care. | Continued until day 30. |
|  | Low protein group | Usual ICU physical exercise |  | Repositioning every 2 hours, usual suction (according to the attending physician's guidance); early passive range of motion training for large joints, once daily, 4-5 times a week, conducted by the nurse team. |  |
| Verceles, 2023 | High protein group | Multimodal rehabilitation program (NMES and additional PT) | Wireless wearable functional electrical stimulation system (Bioness L300 Plus™) | On the basis of standard care, 30 minutes twice daily, up to 5 days per week: 1. One session: Individualized progressive physical therapy (muscle strength and endurance training, focusing on restoring sit to stand and ambulation ability) + 30-minute NMES (applied to bilateral quadriceps and dorsiflexors, parameters as above); 2. The other session: Only 30-minute NMES treatment; NMES continues for 14 days or until ICU discharge, with intensity adjusted to elicit tolerable overt intermittent contractions in sedated patients. | Continued until day 14, or until the patient is discharged from the ICU, whichever comes first. |
|  | Low protein group | Routine physical therapy and rehabilitation (PT) |  | Standard physical and occupational therapy provided by trained hospital therapists according to usual practice of rehabilitation services, including strength and range of motion exercises, and mobility exercises (e.g., sitting at the edge of the bed, sit to stand, ambulation) as tolerated by the patient. |  |
| Liu, 2024 | High protein / Standard protein | Rehabilitative exercise |  | Apart from differing protein dosages, both groups received medical, nursing, and rehabilitative exercise interventions |  |
| Veldsman, 2025 | Common intervention for both groups | Routine mobilization |  | Respiratory physiotherapy and physical rehabilitation, including positioning, range-of-motion exercises, and in-bed mobility; approximately one session daily, initiated on ICU day 3 ± 2 | During ICU stay |
|  | High-protein group | Cycle ergometry (CE) plus routine mobilization | MOTOmed Letto 2 bedside cycle ergometer (RECK-Technik GmbH & Co. KG, Germany) | Semi-recumbent passive or active cycling; started within 1 h after initiation of the intravenous amino acid infusion; 45 min once daily; resistance increased progressively when active cycling was tolerated | Median 6 d (IQR 5–7); at least 5 d, up to ICU day 10 |
|  | Low-protein group | Routine mobilization only | None | Received unit-standard respiratory physiotherapy, positioning, range-of-motion exercises, and in-bed mobility; no additional study cycling intervention | During ICU stay |

| Table S8. Details of nutrition delivery | | |
| --- | --- | --- |
| **Author, year** | **Nutrition prescription** | |
|  | **High protein group** | **Low protein group** |
| Ahmadpour, 2020 | **Protein**:1.5 - 2 g/kg/day  **Calorie**: Standard enteral formula  **Routes**: via nasogastric tube for 5 days | **Protein**: 0.5 - 1.5 g/kg/day  **Calorie**: Standard enteral formula  **Routes**: via nasogastric tube for 5 days |
| Azevedo,2019 | **Protein**: 2.0 - 2.2g/kg/day  **Calorie**: Determined by indirect calorimetry  **Formula**: Peptamen Intense (1.0kcal/mL, 93g/L protein).  **Supplementary nutrition**: Complementary parenteral nutrition if caloric goal not met after 5 days | **Protein**: 1.4 - 1.5g/kg/day  **Calorie**: 25kcal/kg/day  **Formula**: Novasource Senior (1.2kcal/ml, 65g/L protein).  **Supplementary nutrition**: Complementary parenteral nutrition if caloric goal not met after 5 days |
| Azevedo, 2021 | High Protein and Early Exercise (HPE) Group  **Protein**:  - Day 3: 0.8–1.0 g/kg/day - Day 5 onwards: Target 2.0–2.2 g/kg/day  - Day 7: 1.90 (1.7–2.1) g/kg/day  **Calorie**:  - Determined by indirect calorimetry  - Day 3: 50% of measured energy expenditure (MEE)  - Day 5 onwards: 80% of MEE  **Formula:** Peptamen Intense  **Supplementary nutrition**: Complementary parenteral nutrition if caloric goal not met after 7 days | **Protein**:  - Day 3: 0.8–1.0 g/kg/day - Day 5 onwards: Target 1.4–1.5 g/kg/day  - Day 7: 1.34 (1.10–1.45) g/kg/day  **Calorie**:  - determined by indirect calorimetry)  - Day 3: 50% of MEE  - Day 5 onwards: 80% of MEE  **Formula:** Nutritional support as per attending physician and nutrition team  **Supplementary nutrition**: Same as high protein group |
| Badjatia, 2021 | Neuromuscular Electrical Stimulation (NMES) + High Protein (HPRO) group  **Protein**: 1.75 g/kg/day; delivered as enteral bolus of whey protein powder (3 g leucine/feeding, 3 times daily)  **Calorie**: Calculated via Mifflin StJeor (non-intubated) or Penn State (intubated) equations; from oral intake, enteral nutrition, dextrose infusions, and sedatives  **Supplementary nutrition**: Whey protein powder supplementation | Standard of Care (SOC)  **Protein**: 1.2 - 1.4 g/kg/day; via enteral nutritional formulas or specific oral diets  **Calorie**: Same calculation methods as NMES + HPRO group  **Supplementary nutrition**: None permitted |
| Badpeyma, 2025 | **Protein**: Target 2.2 g/kg/day  **Calorie**: Target 25 kcal/kg/day;  **Formula**: BTF (82.26 kcal/100 mL; 5 g protein/100 mL) + ISO whey protein  **Routes**: EN via nasogastric tube ± PN/intravenous amino acids  **Supplementary nutrition**: ISO whey protein; PN if EN inadequate  **Feeding protocol**: Started within 24–48 h of ICU admission; bolus feeding 7 times/day; advanced every 6 h according to tolerance; up to 12 days | **Protein:** Target 1.0 g/kg/day  **Calorie:** Target 25 kcal/kg/day;  **Formula:** Standard BTF without additional whey protein  **Routes:** EN via nasogastric tube ± PN  **Supplementary nutrition:** PN if EN inadequate; no additional protein supplement  **Feeding protocol:** Started within 24–48 h of ICU admission; bolus feeding 7 times/day; advanced every 6 h according to tolerance; up to 12 days |
| Bels, 2024 | **Protein**: 2.0 g/kg/day; 0.10 g protein/mL  **Calorie**: 25 kcal/kg/day; 1.3 kcal/mL  **Formula**: Nutrison Protein Intense  **Supplementary nutrition**: Supplemental parenteral nutrition prohibited in first 7 days, permitted from day 8 onwards  **Feeding protocol**: Started within 48 h of ICU admission at 25% of energy target, increased by 25% daily until 100% on day 4; catch-up feeding from day 5 onwards | **Protein**: 1.3 g/kg/day; 0.06 g protein/mL  **Calorie**: 25 kcal/kg/day; 1.3 kcal/mL  **Formula**: Nutrison Protein Plus  **Supplementary nutrition**: Same as high protein group  **Feeding protocol**: Same as high protein group |
| Bibi,2025 | **Protein**: 2g/kg/day, source: Oral/tube feeds (egg whites-based); parenteral amino acid solutions  **Calorie**: ASPEN guidelines (25-30kcal/kg/day)  **Routes**: Oral, enteral, or parenteral (adjusted to tolerance and medical status) | **Protein**: 1g/kg/day, source: same as high protein group  **Calorie**: ASPEN guidelines (25-30kcal/kg/day)  **Routes**: Same as high protein group |
| Bukhari, 2020* | High-Protein Polymeric Group  **Protein**: 22.4% protein from total calorie  **Calorie**: 25-30 kcal/kg/d  **Formula**: Peptisol  **Routes**: via nasogastric tube, 5 aliquots at 4-hourly intervals daily (24 h) | Oligomeric Group  **Protein**: 16.2% protein from total calorie  **Calorie**: 25-30 kcal/kg/d  **Formula**: Peptamen  **Routes**: Same as High-Protein Polymeric Group |
| Chapple, 2021 | **Formula protein concentration:** 100 g/L  **Calorie**: goal rate achieved within 48 h  **Formula**: Nutrison Protein Intense- 100 g protein/1000 mL; 104 g carbohydrate/1000 mL; fiber-free; protein sources: whey, casein, pea, soy  **Feeding protocol**: 1 mL/kg ideal body weight/h (max 100 mL/h), 24-h continuous delivery; goal rate achieved within 48 h | **Formula protein concentration**: 63 g/L  **Calorie**: goal rate achieved within 48 h  **Formula**: Nutrison Protein Plus- 63 g protein/1000 mL; 142 g carbohydrate/1000 mL; fiber-free; protein sources: whey, casein, pea, soy  **Feeding protocol**: Same as high protein group |
| Danielis, 2019 | Protein-Fortified Diet Group  **Protein**: 1.8 g/kg/day  **Calorie**: Calculated as REE × 0.5 + Protein Intake (REE based on age)  **Formula**: Enteral: 21% protein, 31% carbs/dextrose, 46% fats, 2% fiber; Parenteral: 22% protein, 40% carbs/dextrose, 38% fats | Standard Diet Group  **Protein**: Not specified  **Calorie**: 20-25 kcal/kg/day  **Formula**: Enteral: 16% protein, 35% carbs/dextrose, 49% fats; Parenteral: 16% protein, 49% carbs/dextrose, 35% fats |
| Doig, 2015 | IV Amino Acid Supplement Group  **Protein**: Maximum: 2.0 g/kg/day (including up to 100 g/day IV amino acids from Synthamin 17 Electrolyte Free, plus protein from enteral/parenteral nutrition)  **Calorie**: Determined by attending clinician based on standard ICU practice | Standard Care Group  **Protein**: Protein intake from enteral/parenteral nutrition as per clinician's judgment (no additional IV amino acid supplement)  **Calorie**: Same as IV amino acid supplement group |
| Dresen, 2021 | **Protein**: 1.8 g/kg (BW)/day  **Calorie**: 100% of measured REE (via indirect calorimetry)  **Formula**:  - Non-protein energy: 60% carbohydrates, 40% fat - Preferred route: enteral nutrition; parenteral supplementation for gaps - Additional 24 g protein/day during CRRT | **Protein**: 1.2 g/kg (BW)/day  **Calorie**: 100% of measured REE  **Formula**: Same as high protein group |
| Elkalawy, 2025 | **Protein**: 2.2 g/kg/day  **Calorie**: Indirect calorimetry  **Formula**: High-energy EN formula (1.5 kcal/mL) + enteral protein supplement  **Routes**: Nasogastric/orogastric EN; oral nutrition when conscious  **Feeding protocol**: Started within 24 h; gradually increased to target | **Protein**: 1.5 g/kg/day  **Calorie**: Same as high-protein group  **Formula**: Same enteral formula  **Routes**: Same as high-protein group  **Feeding protocol**: Same as high-protein group |
| Ferrie, 2016 | Higher Amino Acid Group  **Protein**: 1.2 g/kg/day  **Calorie**: 24 kcal/kg/day  **Formula**: Olimel N9 (Baxter): 57 g amino acids/L (21.5% of total energy), 121 g glucose/L (41.1%), 40 g lipids/L (37.4%); osmolarity 1310 mOsm/L | Lower Amino Acid Group  **Protein**: 0.8 g/kg/day  **Calorie**: 26 kcal/kg/day, slightly higher than the higher amino acid group  **Formula**: Oli-Clinomel N7 (Baxter): 40 g amino acids/L (13.3% of total energy), 160 g glucose/L (53.4%), 40 g lipids/L (33.3%); osmolarity 1450 mOsm/L |
| Fetterplace, 2018 | **Protein**: 1.5 g/kg/day  **Calorie**: 25 kcal/kg/day  **Formula**:  - Enteral formula: Nutrison Protein Plus (63 g protein/L, 1250 kcal/L) - Supplements: Beneprotein (6 g protein/scoop)  Feeding protocol: Volume-based | **Protein**: 1.0 g/kg/day  **Calorie**: 25 kcal/kg/day  **Formula**:  - Enteral formula: 1.0 kcal Nutrison (40 g protein/L, 1000 kcal/L)  - No protein supplements  **Feeding protocol**: Hourly-rate-based |
| Heyland, 2023 | **Protein**: ≥2.2 g/kg/day  **Calorie**: use published guidelines  **Feeding protocol**:  - Protein sources: enteral/parenteral nutrition, IV amino acids, or enteral supplements - Duration: up to 28 days, until death, or transition to oral feeding - Target achievement: 80% of prescribed dose | **Protein**: ≤1.2 g/kg/day  **Calorie**: same in both groups  **Feeding protocol**: Same as high protein group |
| Kagan, 2022* | High protein + cycle  **Protein**: Actual delivery- 83.7 ± 31.9 g/day  **Calorie**: 80% of measured REE  **Formula**: Promote: 14.8 g protein/237 mL, 1.0 Cal/mL, 25% protein (kcal), 50% carbohydrate (kcal), 25% fat (kcal)  **Feeding protocol**: EN delivered via nasogastric/orogastric tube; tolerance assessed by GRV every 8 h | low protein + cycle  **Protein**: Actual delivery- 67.2 ± 20.2 g/day  **Calorie**: 80% of measured REE  **Formula**: Jevity: 10.4 g protein/237 mL, 1.06 Cal/mL, 16.7% protein (kcal), 54.3% carbohydrate (kcal), 29.0% fat (kcal)  **Feeding protocol**: Same as high protein group |
| Liu, 2024 | **Protein**: 1.8g/kg/d  **Calorie**: 25kcal/kg/d (first 10 days in ICU) | **Protein**: 1.2g/kg/d  **Calorie**: 25kcal/kg/d (first 10 days in ICU) |
| Miri, 2025 | **Protein:** 1.5 g/kg/day **Calorie:** NA **Routes:** EN via nasoenteric tube **Formula:** High-protein enteral formula **Supplementary nutrition:** PN was not routinely prescribed as part of the intervention; the need for PN or a change in nutrition-delivery route was determined by treating physicians **Feeding protocol:** Initiated after randomization. Continued until ICU discharge, death, extubation, change in nutrition-delivery route, or day 21 | **Protein:** 1.0 g/kg/day **Calorie:** NA **Routes:** EN via nasoenteric tube **Formula:** Standard-care enteral nutrition protocol;  **Supplementary nutrition:** Same as high-protein group **Feeding protocol**: Same as high-protein group |
| Nakamura, 2021 | **Protein**: 1.8 g/kg/day  **Calorie**: 20 kcal/kg/day  **Formula**: Peptamen Intense (containing 9.2 g whey protein and 100 kcal per 100 ml)  **Feeding protocol**: Continuously via nasogastric tube, starting at 10-20 ml/h, increased by 10-20 ml/h every 8 hours if tolerated (based on gastric residual volume). PN was used as adjunct if EN was insufficient. | **Protein**: 0.9 g/kg/day  **Calorie**: 20 kcal/kg/day  **Formula**: Glucerna REX (containing 4.2 g soy protein and 100 kcal per 100 ml)  **Feeding protocol**: Same as high protein group |
| O'Keefe, 2025 | **Protein**: Target total protein 2.0 g/kg/day **Calorie**: 25 kcal/kg/day **Formula**: Standard polymeric EN, primarily Replete (1.0 kcal/mL; 62.4 g protein/L; 25% kcal from protein) + ProSource **Routes**: Continuous EN via orogastric/nasogastric tube; ProSource boluses 60–180 mL, 2–4 times/day **Supplementary nutrition**: Enteral protein supplementation with ProSource **Feeding protocol**: Started at 20 mL/h and advanced over 24 h; supplement reduced after ≥75% caloric target and adjusted to maintain total protein at 2.0 g/kg/day | **Protein**: Standard EN without additional protein supplementation  **Calorie**: 25 kcal/kg/day **Formula**: Standard polymeric EN, primarily Replete (1.0 kcal/mL; 62.4 g protein/L; 25% kcal from protein) **Routes**: Continuous EN via orogastric/nasogastric tube **Supplementary nutrition**: None **Feeding protocol**: Started at 20 mL/h and advanced over 24 h to the caloric target |
| Opoda, 2024 | **Protein**: 2 g/kg/day  **Calorie**: 20-25 kcal/kg/day  **Formula**: Standard nutrition formula + 60 g protein  **Feeding protocol**: EN preferred, initiated within 48 h of ICU admission at 20 mL/h, titrated to meet energy goal. Enteral or parenteral amino acid supplements used to reach protein target. | **Protein**: 1.2 g/kg/day  **Calorie**: 20-25 kcal/kg/day  **Formula**: Same as high protein group  **Feeding protocol**: Same as high protein group |
| Parasuraman,2025 | **Protein**: 1.5 g/kg/day  **Calorie**: 25 kcal/kg/day  **Feeding protocol**: Enteral feeding initiated within 24 hours of ICU admission. The diet is prepared by the treating nurse by adding extra protein powder to the standard feed. | **Protein**: 1 g/kg/day  **Calorie**: 25 kcal/kg/day  **Feeding protocol**: Enteral feeding started within 24 hours of ICU admission. The standard feed is prepared by the dietician according to the study's requirements. |
| Reignier, 2023 | **Protein**:  - Days 1-7: 1.0–1.3 g/kg/day - From day 8 onwards: 1.2–2.0 g/kg/day  **Calorie**:  - Days 1-7: 25 kcal/kg/day - From day 8 onwards: 30 kcal/kg/day  **Feeding protocol**: Bedside physicians determined the optimal feeding route (enteral or parenteral) daily to meet calorie targets | **Protein**:  - Days 1-7: 0.2–0.4 g/kg/day - From day 8 onwards: 1.2–2.0 g/kg/day  **Calorie**:  - Days 1-7: 6 kcal/kg/day - From day 8 onwards: 30 kcal/kg/day  **Feeding protocol**: Same as high protein group |
| Rugeles, 2013 | **Protein**: 1.7 g/kg/day, Actual delivery- 1.4 g/kg/day  **Calorie**: 15 kcal/kg/day  **Formula**: 1300 kcal/L, 66.6 g protein/L plus twice-daily soy protein modules to reach protein target. Maintained for 7 days, then switched to standard regimen (25 kcal/kg/day without protein boluses) if needed.  **Route**: Continuous enteral feeding | **Protein**: 20% of calories from protein, Actual delivery- 0.76 g/kg/day  **Calorie**: 25 kcal/kg/day  **Formula**: the same base formula as the hyperproteic group (no additional protein modules)  **Route**: Continuous enteral feeding |
| Singer, 2007 | High-dose Amino Acids  **Protein**: 150 g/day  **Calorie**: 2000 non-protein kcal/day  **Formula**: At twice the dose of normal -dose amino acids group  **Route**: via PN | Normal-dose Amino Acids  **Protein**: 75 g/day  **Calorie**: 2000 non-protein kcal/day  **Formula**: Non-protein calories from dextrose and Intralipid; amino acid formulation is 10% Aminoplasmal (B Braun, Germany)  **Route**: via PN |
| Summers, 2025 | **Formula protein concentration**: 100 g protein/L  **Calorie**: Same in both groups  **Formula**: Nutrison Protein Intense  **Route**: EN  **Feeding protocol**: Formula administered sequentially for 3-month blocks over 12 months. | **Formula protein concentration**: 63 g protein/L  **Calorie**: Same in both groups  **Formula**: Nutrison Protein Plus  **Route**: EN  **Feeding protocol**: Same as high protein group |
| Uyar, 2023 | **Protein**: 2.0 g/kg/day  **Calorie**: 25 kcal/kg/day  **Formula**: Nutrition Protein Plus (63 g protein/L) plus protein supplements (Potiphar, 2.2 g protein/scoop)  **Feeding protocol**: Targets reached gradually within 4 days with continuous enteral feeding. | **Protein**: 1.2 g/kg/day  **Calorie**: 25 kcal/kg/day  **Formula**: Standard enteral formula (1.0 kcal Nutrison, 40 g protein/L)  **Feeding protocol**: Targets reached gradually within 4 days with continuous enteral feeding. |
| van Zanten, 2018 | **Protein**:8 g/100 kcal  **Calorie**: 25 kcal/kg (IBW)/day  **Formula**: Enteral formula (1.25 kcal/ml)  **Feeding protocol**: via nasogastric tube, starting at 20 ml/h and increasing by 20 ml/h to reach target. For BMI > 30 kg/m², IBW (30 × height² in meters) was used for target calculation. Parenteral nutrition allowed if needed. | **Protein**: 5 g/100 kcal  **Calorie**: 25 kcal/kg (IBW)/day  **Formula**: Nutrison Protein Plus  **Feeding protocol**: Same as high protein group |
| Veldsman, 2025 | **Protein:** Standard target 1.3 g/kg/day + intravenous amino acids 0.25–0.40 g/kg/day **Calorie:** IC-guided; if unavailable, ≤20–25 kcal/kg/day to day 7, then 25–30 kcal/kg/day **Formula:** Standard EN/PN formula + Vamin 18 electrolyte free **Routes:** EN ± PN; additional amino acids via central venous catheter **Supplementary nutrition:** PN if EN target not achieved by day 4 **Feeding protocol:** Standard nutrition started early and advanced over 72 h; IV amino acids started on day 3–4, infused over 4 h daily for ≥5 days, up to day 10 | **Protein**: 1.3 g/kg/day **Calorie**: IC-guided; if unavailable, ≤20–25 kcal/kg/day to day 7, then 25–30 kcal/kg/day **Formula**: Standard EN/PN formula **Routes**: EN ± PN **Supplementary nutrition**: PN if EN target not achieved by day 4 **Feeding protocol**: Standard nutrition started early and gradually advanced over the first 72 h |
| Verceles, 2023 | NMES+HPRO+PT  **Protein**: 1.75 g/kg/day  **Calorie**: Calculated by ICU dietician  **Formula**: Received usual nutrition plus powdered whey protein supplements (Optimum Nutrition) to meet protein targets, administered via enteric access with 40 mL water | Standard Care (SC)  **Protein**: Based on ASPEN guidelines  **Calorie**: Calculated by ICU dietician  **Formula**: Received usual enteral/parenteral nutrition based on standard protocols, with protein from regular formulas and no additional supplements |
| Wang, 2024 | **Protein**:  - Days 1-3: 1.5 g/kg/day  - From Day 4 onwards: 1.5 g/kg/day  **Calorie**:  - Days 1-3: 20 kcal/kg/day (non-protein calories) - From Day 4 onwards: 25 kcal/kg/day (non-protein calories)  **Route**: EN  **Feeding protocol**: Initial rate: 10–15 mL/h, with tolerance assessed every 4 hours and rate adjusted incrementally if tolerated | **Protein**:  - Days 1-3: 0.8 g/kg/day  - From Day 4 onwards: 1.5 g/kg/day  **Calorie**:  - Days 1-3: 20 kcal/kg/day (non-protein calories) - From Day 4 onwards: 25 kcal/kg/day (non-protein calories)  **Route**: EN  **Feeding protocol**: Same as high protein group |
| Zhang, 2022 | **Protein**: 2.0 g/kg/d  **Calorie**: 25-30 kcal/kg/d  **Formula**: Commercial enteral formula + 100% whey protein powder supplements (8.16 g protein/bag)  **Route**: via nasogastric tube | **Protein**: 1.2 g/kg/d  **Calorie**: 25-30 kcal/kg/d  **Formula**: Commercial enteral formula (protein fiber-type nutrient suspension)  **Route**: via nasogastric tube |

Note：BTF, blenderized tube feeding; ASPEN, American Society for Parenteral and Enteral Nutrition; EN, enteral nutrition; PN, parenteral nutrition; RME, resting metabolic expenditure; REE, resting energy expenditure; MEE，measured energetic expenditure; BW, body weight; GRV, gastric residual volume; IBW, ideal body weight; NMES, neuromuscular electrical stimulation; HPRO, High Protein supplementation group; IC, indirect calorimetry; PT, physical therapy.

*Bukhari 2020 has 3 groups: control, high-protein polymeric and oligomeric group, the control group was excluded from the analysis.

*Kagan 2022 has 3 groups: Group 1 (control), Group 2 (low protein + cycle), and Gorup 3 (high protein + cycle). Group 1 was excluded from the analysis.

Table S9: Sensitivity analysis of Mortality

1. Sensitivity analyses of all-cause mortality according to protein-delivery cut-offs

**Panel A:** Sensitivity analysis using 1.2 g/kg/day as the cut-off to distinguish the higher- and lower-protein groups.

**Panel B:** Sensitivity analysis using 1.3 g/kg/day as the cut-off to distinguish the higher- and lower-protein groups.

**Panel C:** Sensitivity analysis using 1.5 g/kg/day as the cut-off to distinguish the higher- and lower-protein groups.

Table S9: Sensitivity analysis of Mortality (Isocaloric Trials using the same energy target or prescription strategy by design)

Table S10. Publication bias for 4 risk factors.

| **Risk factors** | **Egger’s test** | | **Begg’s test** | | **Trim-and-fill analyses** | |
| --- | --- | --- | --- | --- | --- | --- |
|  | **t** | **p** | **z** | **p** | **Imputed studies** | **Adjusted effect** |
| Mortality | -1.83 | 0.0799 | -1.17 | 0.24 | 6 | RR 1.06, 95% CI (0.92; 1.23) |
| Duration of MV | -2.30 | 0.0330 | -0.24 | 0.81 | 9 | MD 0.39, 95% CI (-0.34; 1.12) |
| ICU length of stay | -2.55 | 0.0184 | 0.84 | 0.40 | 6 | MD 0.32, 95% CI (-0.42; 1.06) |

Note: MV, mechanical ventilation; ICU, intensive care unit.

Figure S1. Assessment on risk of bias for included RCTs

Figure S2. Funnel plot for included studies

Note: A, Funnel plot of mortality. B, Funnel plot of MV length of stay. C, Funnel plot of ICU length of stay.

Figure S3. 28/30-, 60-, 90-, 180-day, ICU, Hospital Mortality analysis for included RCTs.

Figure S4. ICU length of stay (LOS), TSA, and subgroup analysis for included RCTs

Note: A, ICU LOS. B, Trial sequential analysis (TSA) of ICU LOS. C, subgroup analysis of ICU LOS. DARIS: diversity adjusted required information size is the calculated optimum sample size for statistical inference, MID: minimally important difference.

Figure S5. Duration of MV, TSA, and subgroup analysis for included RCTs

Note: A, Duration of MV. B, Trial sequential analysis (TSA) of duration of MV. C, subgroup analysis of duration of MV. DARIS: diversity adjusted required information size is the calculated optimum sample size for statistical inference, MID: minimally important difference.

Figure S6. Atrophy rate of thigh muscle thickness, thigh muscle cross sectional area (CSA), diaphragm and mid-upper arm circumference analysis for included RCTs

Note: A, Atrophy rate of thigh muscle thickness and subgroup analysis. B, Atrophy rate of thigh muscle cross sectional area (CSA) and subgroup analysis. C, Atrophy rate of diaphragm. D, Absolute change in mid-upper arm circumference.

Figure S7. Handgrip strength and HRQoL analysis for included RCTs

Note: A, Handgrip strength. B, HRQoL.

Figure S8. Biochemical tests analysis for included RCTs

Note: A, Nitrogen balance. B, Albumin. C, Urea. D, Creatinine. E, Blood urea nitrogen (BUN).

Figure S9. Adverse effects analysis for included RCTs

Note: A, Feeding intolerance. B, Diarrhea. C, Regurgitation / vomiting. D, Constipation. E, Receipt of promotility agents. F, Received RRT / Incident RRT. G, Infection in ICU.

Figure S10 Sensitivity analysis: studies with the same prescribed energy target in both groups.

1) 28/30-day Mortality

2)60-day Mortality

3)90-day Mortality

4)180-day Mortality

5)ICU Mortality

6) Hospital Mortality

7) Duration of MV

8) Subgroup analysis of duration of MV

9) ICU LOS

10) Subgroup analysis of duration of ICU LOS

11) Atrophy rate of thigh muscle thickness

12) Subgroup analysis of atrophy rate of thigh muscle thickness

13) Atrophy rate of thigh muscle CSA

14) Subgroup analysis of atrophy rate of thigh muscle CSA

15)) Atrophy rate of diaphragm

16) Handgrip strength

17) HRQoL

18) Nitrogen balance

19) Albumin

20) Urea

21) Creatinine

22) BUN

23) Feeding intolerance

24) Diarrhea

25) Regurgitation or vomiting

26) Received RRT/Incident RRT

27) Infection in ICU

Figure S11. The GRADE assessment of the certainty of the evidence

| Quality assessment | | | | | | | Summary of Findings | |
| --- | --- | --- | --- | --- | --- | --- | --- | --- |
| Participants (studies) | Risk of bias | Inconsistency | Indirectness | Imprecision | Publication bias | Overall quality of evidence | Study event rates (%) | |
|  |  |  |  |  |  |  | Low protein | High protein |
| ICU LOS | | | | | | | | |
| 10564 (24 RCTs) | not serious^1^ | serious^2^ | not serious | serious^3^ | undetected | ⊕⊕⊝⊝  **LOW**^1,2,3^ | 5311 | 5253 |
| MV duration | | | | | | | | |
| 9992 (21 RCTs) | not serious^4^ | serious^2^ | not serious | not serious | undetected | ⊕⊕⊕⊝  MODERATE^2,4^ | 5029 | 4963 |
| Atrophy rate of thigh muscle thickness | | | | | | | | |
| 601 (7 RCTs) | serious^5^ | serious^2^ | not serious | not serious | undetected | ⊕⊕⊝⊝  **LOW**^2,5^ | 298 | 303 |
| Atrophy rate of thigh muscle CSA | | | | | | | | |
| 469 (7 RCTs) | not serious^6^ | not serious | not serious | not serious | undetected | ⊕⊕⊕⊕  HIGH ^6^ | 234 | 235 |
| Atrophy rate of diaphragm | | | | | | | | |
| 210 (3 RCTs) | serious^7^ | serious^2^ | not serious | serious^8^ | undetected | **⊕⊝⊝⊝**  **VERY LOW**^2,7,8^ | 105 | 105 |
| Handgrip strength | | | | | | | | |
| 230 (3 RCTs) | not serious^9^ | not serious | not serious | serious^8^ | undetected | ⊕⊕⊕⊝  MODERATE^8,9^ | 117 | 113 |
| HRQoL | | | | | | | | |
| 1082 (3 RCTs) | not serious^9^ | not serious | not serious | not serious | undetected | ⊕⊕⊕⊕  HIGH^9^ | 545 | 537 |
| Nitrogen balance | | | | | | | | |
| 476 (9 RCTs) | serious^10^ | serious^2^ | not serious | not serious | undetected | ⊕⊕⊝⊝  **LOW** ^2,10^ | 242 | 234 |
| Albumin | | | | | | | | |
| 1310 (6 RCTs) | serious^11^ | not serious | not serious | not serious | undetected | ⊕⊕⊕⊝  MODERATE ^11^ | 641 | 669 |
| Urea | | | | | | | | |
| 2379 (5 RCTs) | not serious^12^ | not serious | not serious | not serious | undetected | ⊕⊕⊕⊕  HIGH^12^ | 1184 | 1195 |
| Creatinine | | | | | | | | |
| 2194 (8 RCTs) | serious^13^ | not serious | not serious | not serious | undetected | ⊕⊕⊕⊝  MODERATE ^13^ | 1107 | 1087 |
| BUN (blood urea nitrogen) | | | | | | | | |
| 777 (5 RCTs) | serious^14^ | not serious | not serious | not serious | undetected | ⊕⊕⊕⊝  MODERATE ^14^ | 390 | 387 |
| Feeding intolerance | | | | | | | | |
| 1094 (4 RCTs) | not serious^15^ | serious^2^ | not serious | serious^16^ | undetected | ⊕⊕⊝⊝  **LOW**^2,15,16^ | 50/545  (9.2%) | 59/549  (10.7%) |
| Diarrhea | | | | | | | | |
| 3868 (6 RCTs) | serious^17^ | not serious | not serious | serious^3^ | undetected | ⊕⊕⊝⊝  **LOW** ^3,17^ | 608/1942 (31.3%) | 690/1926 (35.8%) |
| Regurgitation or vomiting | | | | | | | | |
| 3808 (5 RCTs) | serious^18^ | not serious | not serious | serious^3^ | undetected | ⊕⊕⊝⊝  **LOW** ^3,18^ | 349/1912 (18.3%) | 423/1896 (22.3%) |
| Constipation | | | | | | | | |
| 3080 (2 RCTs) | not serious^19^ | not serious | not serious | serious^3^ | undetected | ⊕⊕⊕⊝  MODERATE^3,19^ | 436/1543 (28.3%) | 451/1537 (29.3%) |
| Receipt of promotility agents | | | | | | | | |
| 4081 (3 RCTs) | not serious^15^ | not serious | not serious | serious^3^ | undetected | ⊕⊕⊕⊝  MODERATE^3,15^ | 325/2042 (15.9%) | 481/2039 (23.6%) |
| Received RRT / Incident RRT | | | | | | | | |
| 8806 (8 RCTs) | not serious^15^ | not serious | not serious | serious^3^ | undetected | ⊕⊕⊕⊝  MODERATE^3,15^ | 676/4419 (15.3%) | 701/4387 (16.0%) |
| Infection in ICU | | | | | | | | |
| 4724 (7 RCTs) | serious^20^ | serious^2^ | not serious | serious^3^ | undetected | ⊕⊝⊝⊝  **VERY LOW**^2,3,20^ | 471/2367 (19.9%) | 473/2357 (20.0%) |
| Mortality | | | | | | | | |
| 11099  (26 RCTs) | Serious^21^ | not serious | not serious | not serious | undetected | ⊕⊕⊕⊝  MODERATE^21^ | 1740/5580  (31.2%) | 1758/5519 (31.9%) |
| Absolute change in mid-upper arm circumference | | | | | | | | |
| 194  (3 RCTs) | not serious^22^ | serious^2^ | not serious | serious^8^ | undetected | ⊕⊕⊝⊝  **LOW**^2,8,22^ | 98 | 96 |

^1^ Only 5/24 (20.8%) trials were high risk of bias.

^2^ Inconsistency index is > 50%
^3^ Required information size(RIS) was not reached
^4^ 11/21 (52.4%) trials were low risk of bias, 6/21 (28.6%) trials was some concerns and 4/21 (19%) trials was high risk of bias
^5^ Only 2/7 (28.6%) trials were low risk of bias
^6^ Only 2/7 (28.6%) trial were high risk of bias
^7^ 2/3 (66.7%) trials were some concerns and 1/3 (33.3%) trial was high risk of bias

^8^ Magnitude of the sample is less than 400 (recommended informative sample size for continuous outcomes)
^9^ 2/3 (66.7%) trials were low risk of bias and 1/3 (33.3%) trial was some concerns
^10^ 2/9 (22.2%) trials were low risk of bias, 3/9 (33.3%) trials were some concerns and 4/9 (44.4%) trials were high risk of bias
^11^ 2/6 (33.3%) trials were low risk of bias and 4/6 (66.7%) trials were some concerns
^12^ 3/5 (60%) trials were low risk of bias, 1/5 (20%) trial was some concerns, and 1/5 (20%) trial was high risk of bias

^13^ Only 2/8(25%) trials were low risk of bias.
^14^ 1/5 (20%) trial was low risk of bias, 1/5 (20%) trials was some concerns and 3/5 (60%) trial were high risk of bias

^15^ The largest study had low risk of bias
^16^ TSA revealed that too little information of the RIS were attained
^17^ 3/6 (50%) trials were low risk of bias and 2/6 (33.3%) trials were some concerns, and 1/6 (16.7%) trials were high risk of bias.

^18^ 2/5 (40%) trials were low risk of bias, 2/5 (40%) trial were some concerns, and 1/5 (20%) trial was high risk of bias
^19^ 50% trials were low risk of bias and 50% trials were some concerns
^20^ 1/7 (14.3%) trial was low risk of bias, 4/7 (57.1%) trials were some concerns and 2/7 (28.6%) trial were high risk of bias

^21.^ 11/26 (42.3%) trials were low risk of bias, 7/26 (26.9%) trials were some concerns, and 8/26 (30.8%) trials were high risk of bias

^22^ 2/3(66.7%) trials were low risk of bias
